# Predicting badger visits to farm yards and making predictions available to farmers

**DOI:** 10.1371/journal.pone.0216953

**Published:** 2019-05-24

**Authors:** Andrew Robertson, Joanna Judge, G. J. Wilson, Ian J. Vernon, Richard J. Delahay, Robbie A. McDonald

**Affiliations:** 1 Environment and Sustainability Institute, University of Exeter, Penryn Campus, Penryn United Kingdom; 2 National Wildlife Management Centre, Animal and Plant Health Agency, Woodchester Park, Nympsfield, United Kingdom; 3 National Biodiversity Network, Nottingham, United Kingdom; 4 Biocensus Limited, Bath, United Kingdom; Bangor University, UNITED KINGDOM

## Abstract

The use of agricultural resources or environments by wildlife may result in opportunities for transmission of infections amongst wild animals, livestock and humans. Targeted use of biosecurity measures may therefore reduce disease risks, although this requires practical knowledge of where such measures would be most effective, and effective means of communicating risks so that stakeholders can make informed decisions about such investment. In parts of Europe, the European badger *Meles meles* may act as a wildlife reservoir for *Mycobacterium bovis*, the causative agent of bovine tuberculosis, and badger visits to farmyards may provide potential opportunities for transmission of *M*. *bovis* to cattle. Biosecurity measures are effective in reducing badger activity in farmyards, although it is unclear which farms should be targeted with such measures. We used cameras to monitor badger activity in 155 farmyards in south west England and Wales, and related variations in the presence and frequency of badger visits to farm characteristics. Badgers were recorded on camera in 40% of farmyards monitored. However, the frequency of visits was highly variable, with badgers recorded on >50% of nights in only 10% of farms. The presence of badgers in farmyards was positively associated with the density of badger setts, the number of feed stores and the number of cattle sheds, and negatively associated with the distance to the nearest active badger sett, the presence of a house/dwelling and the number of cattle housed on the farm. The frequency of visits was negatively associated with the distance to the nearest active badger sett and the number of cattle housed. Models predicted the presence/absence of badgers in farmyards with 73% accuracy (62% sensitivity, 81% specificity, using a cut off value of 0.265). Models could not distinguish between farms with low/high frequency of visits, although farms predicted as having badgers present typically had a higher frequency of visits than those that were not. We developed and present an interactive web based application: the Badger Farm Assessment Tool (BFAT), to allow users to enter the characteristics of a farm and generate a relative risk score describing the likelihood of badger visits.

## Introduction

Wildlife species act as reservoirs for many globally important and emerging diseases of humans and livestock [1, 2]. Management of diseases in wildlife may involve reducing host numbers, vaccination, medication or modifying the environment in order to reduce opportunities for disease transmission [3, 4]. Interventions in wildlife populations are challenging and often costly, but where wild animals exploit anthropogenic resources, opportunities exist to take practical steps to manage access and hence reduce the risks of infection transmission. In order to evaluate and install such measures effectively, we require information on patterns of resource exploitation by the target wildlife species.

Bovine tuberculosis (bTB), caused by *Mycobacterium bovis*, is a globally important disease of cattle, and in many countries wild animals can act as a reservoir and a potential source of infection for livestock [5]. In the United Kingdom and Ireland, European badgers (*Meles meles*) are the principal wildlife reservoir of *M*. *bovis* infection, where they contribute to varying extents, to the ongoing cattle bTB epidemic [6–8]. Infected badgers may excrete *M*. *bovis* in their urine, saliva or faeces and it has been suggested that onward transmission to cattle could potentially occur either directly during close contact, or indirectly via contamination of the environment [9]. Farmyards have been suggested as potentially important location for transmission, as several studies have recorded badgers entering yards and farm buildings, with observed instances of close direct contact with cattle and contamination of cattle feed, bedding or water with excretory products [10–17]. Experimental studies have shown that *M*. *bovis* can survive in substrates including feed, water or soil [18] and that environmental contamination by deer can result in infection in cattle [19]. For these reasons, wildlife activity in farmyards is a concern for bTB control and is the subject of ongoing research in the UK and several other countries where there is a transmission risk from wildlife [16, 20–23].

Experimental trials have shown that practical measures such as sheet gates or electric fencing can be used to reduce badger activity in farmyards [14, 24, 25]. Judge et al. [14] demonstrated that such measures were 100% effective in keeping badgers out of farmyards, when correctly deployed and adequately maintained, and could significantly reduce activity, even when applied to only part of the farm (feed stores or cattle housing). In a review of the potential transmission pathways and management options, Ward et al. [26] suggested that excluding badgers from farmyards or buildings may be a simple and effective strategy for reducing the risk of bTB transmission to cattle, and current UK government advice to farmers recommends the use of such measures as part of a ‘five point plan’ for biosecurity (http://www.tbhub.co.uk/biosecurity/protect-your-herd-from-tb/). However, although badgers have been observed in farmyards in multiple studies in south-west England [10, 12, 14], recent studies from both England and Ireland have reported relatively little activity in farmyards [16, 17, 22], suggesting that this behaviour may be less frequent in certain situations. In addition, significant variation in badger visits have been observed among farms within studies [14, 17]. Research by [14] recorded no badger activity at 41% of farms monitored for 12 months, in an area of the UK with generally high badger densities and high incidence rates of bTB in cattle.This study also found substantial variation in the frequency of visits amongst the 59% of farms that did experience badger visits, with only a handful of sightings at some farms, but sightings on more than 60% of nights at around 10% of farms. The reasons for this variation are unclear, although a recent meta-analysis using data from five studies, indicated that badger population density was positively correlated with badger visits to farmyards [17]. Unfortunately the relatively small scale of studies to date ([10] = 2 farms, [14] = 40 farms, [17] = 20 farms), has made it difficult to investigate this variation further.

Applying biosecurity measures to farms can potentially be expensive depending on the measures used (approximately £650 - £12500) and should ideally deployed in cost effective way to farms with badger activity. Information is therefore required on the extent of badger visits to farmyards at the landscape scale, and on the factors which influence the likelihood that visits occur. In addition, tools are needed to effectively communicate such assessments to stakeholders (farmers or vets) so that they can make informed decisions on whether and how to implement biosecurity measures.

We used motion activated surveillance cameras to monitor badger activity in buildings and yards on 155 farms in the south west of the UK. We investigated whether the presence of badgers and the levels of activity observed were related to farm characteristics, and whether such relationships could be used to predict this activity. Finally, we used the statistical relationships identified to build an interactive web-based tool to allow stakeholders to enter the characteristics of a farm and generate a relative score of the likelihood of badger visits.

## Materials and methods

### 2.1 Farm selection

**Fig 1:**
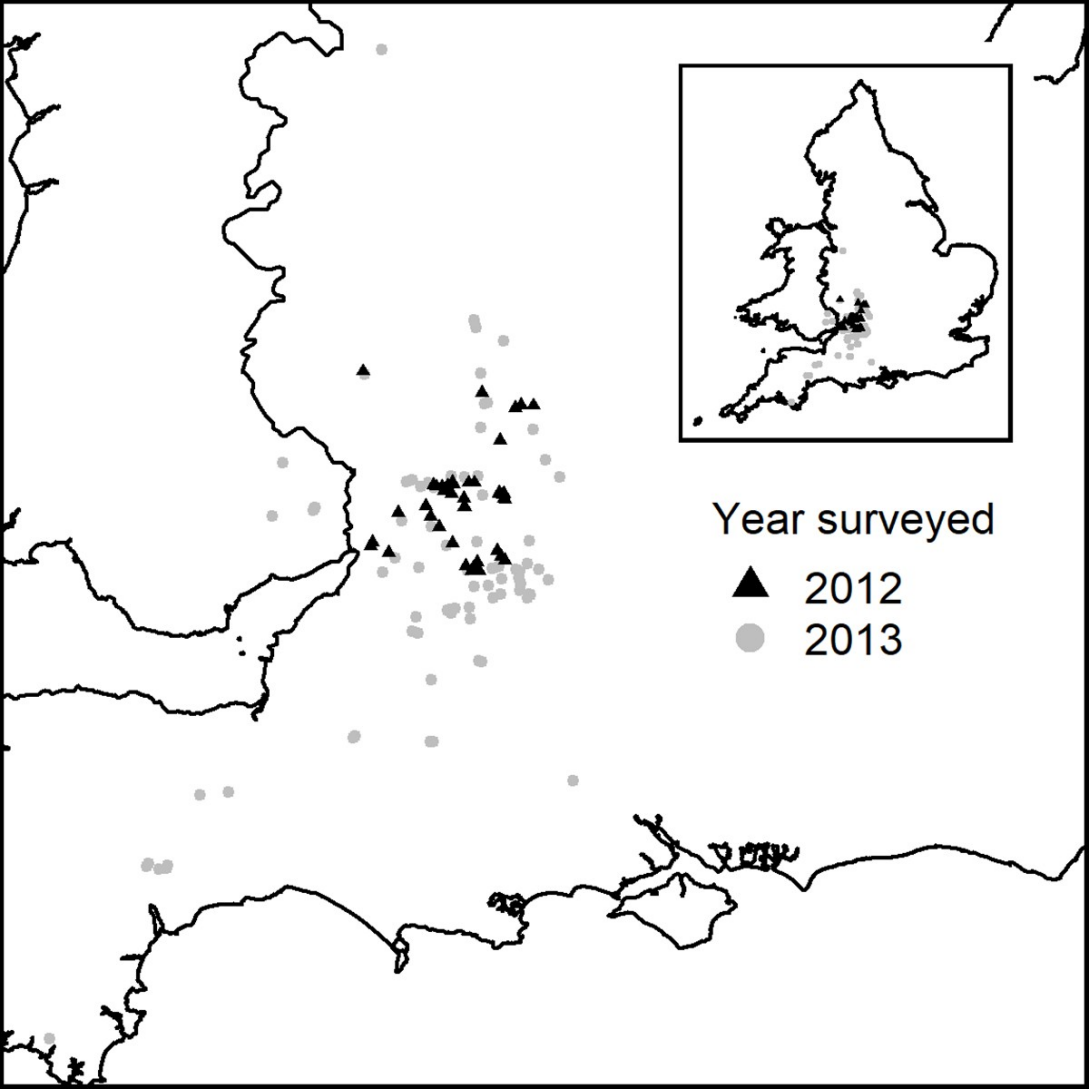
The location of farms included in the present study where badger surveys and monitoring work were undertaken.

The study was carried out on 155 farms in 2012 and 2013, comprising 75 in Gloucestershire, 75 in the wider south-west of England and five in Wales (Fig 1), all in the high risk disease regions in GB. Farms were recruited into the study by either a personal visit, word of mouth, or via adverts placed in the local farming press. All farms had a cattle herd under annual bTB testing and kept cattle or cattle feed in buildings for at least part of the year. As farms were not randomly selected, it is possible that there is a bias in the sample such that farms are not representative of those in the wider landscape. For example, those with TB, or suspected badger activity may have been more likely to join the study. The implications of this are discussed in more detail in the discussion section.

### 2.2 Farm surveys

#### 2.2.1 Badger surveys

All accessible land within a 500 m radius of the approximate central point of each farm yard was surveyed for badger setts (communal burrows), and the location and activity level (number of well used, partially used and unused entrance holes) was recorded for each sett. A 500 m radius from the farm yard was chosen because this approximates to the size of badger social group territories observed and estimated for south-west England (social group density is around one per 0.8 km^2^ [27, 28]). If badgers were present in the area and accessing the farm then it is likely that at least some setts would fall within this radius. It is possible that this area could encompass setts resident on the farm, or an intersection of groups from neighbouring land. This survey data therefore provides a relative index of badger activity in the immediate vicinity surrounding each farm. In some instances, it was not possible to survey all land within the 500 m radius. Where possible, badger setts located over field boundaries from adjacent land that was accessible were noted; however, it was not possible to record activity scores for those setts. This is typical of many farms in the landscape where all land is not accessible to famers or surveyors. In order to control for this the total area (in km^2^) was calculated for each farm.

#### 2.2.2 Camera surveys

Badger activity in farmyards was recorded using infra-red, motion triggered camera traps (Bushnell Trophy Cam, Bushnell Outdoor Products, Overland Park, Kansas, USA). Cameras were deployed at potential badger access points to cattle sheds, feed stores, hay/straw barns, silage clamps and yards on all study farms. Between two and nine cameras were deployed on each farm, depending on the size and the number of buildings and potential entrance points for badgers. The cameras were set to take still images (a burst of three when triggered) and were operational for 24 hours each day throughout the surveillance period on each farm. Cameras were in place for a minimum of 4 weeks at each farm (range 28–39 days, median = 30). Camera surveillance was carried out on 75 farms from 11th April to 24th September 2012 (Year 1), and on a further 80 farms from 17th April to 30th September 2013 (Year 2). Surveillance was carried out from April to September as previous studies have shown that badger visits are generally low in winter, and are more frequent to in spring and summer, particularly in drier conditions [12]. Given limitations on the availability of staff and cameras, the surveillance was carried out in a rolling program throughout the surveillance period. Each camera was fitted with a memory card (2 Gb minimum) and was visited half-way through the surveillance period on each farm to check battery life, memory card space and any camera malfunctions or damage. After the cameras were collected, digital photographs were downloaded and checked for images of badgers. The dates and times of all images of badgers were collated on a database for each camera, along with the type of building visited (i.e. feed store, silage clamp or cattle housing).

#### 2.2.3 Collection of other farm level variables

Data were collected on several variables relating to the potential attractiveness of farms to badgers, either by direct observation or discussions with farmers. Survey variables were chosen based on the published literature [10, 12, 14, 27] and expert opinion (the authors and other badger ecologists with the Animal and Plant Health Agency). Efforts were also made to choose variables that would be easy to collect (i.e. questions that farmers are able to answer easily), such that they could potentially be used in future farm assessment surveys. Four variables related to food availability; number of feed stores; production of palatable crops; cattle feed type; and feed accessibility (Table 1). Three variables related to farm size and type; number of cattle sheds; maximum number of housed cattle and presence of a dairy (milking parlour) on the farm (Table 1). Two variables related to human presence or disturbance; presence of a house; and presence of lights (Table 1). The presence of dogs on the farm (yes/no answer from asking the farmer) was also included as a variable, as dogs may potentially deter wildlife. Unfortunately it was not possible to determine whether dogs were roaming at night or to quantify dog presence on camera from the data available. Due to the rolling nature of the surveillance program, season was also included as three level categorical variable (spring = April–May, summer = June–July, autumn = August–September) to account for potential changes in badger behaviour and diet over the survey period [27]. Previous studies have shown that badger visits to farmyards peak in dry conditions in summer, likely due reduced availability of invertebrate prey [12]. To account for this we also calculated the proportion of nights (over the observation period) which were classed as ‘worm nights’ following [29]. A worm night (ie a night when worms are available to badgers) is classed as a night where the temperature did not fall below 0°C and where there was at least 2mm of rain in the preceding 72 hours [29]. Night length potentially determines the length of observation period for each night, with more opportunity for activity when nights are longer. However, night length was not included in analyses, as this was partly explained by the season variable. In addition, previous observations that badger visits to farmyards are higher in summer (when nights are shorter), suggests that longer nights (observation periods) do not increase opportunities for badger activity in farmyards.

**Table 1:** Description of farm level variables recorded during the present study.

Category	Variable	Description	Type
Badger field signs	Badger sett density	Number of badger setts recorded divided by area surveyed	Continous
	Nearest active sett	Distance from farm buildings/yard to nearest active badger sett, to nearest 100m (0–100m, 101–200m, 201–300m, 301–400m, 401–500 m, >500 m)	Continuous (1–6)
Farm Characteristics	Feed Stores	Number of feed stores on farm	Continuous
	Palatable crops	Does the farmer grow crops palatable to badgers? (cereals; wheat, barley, maize, oats)	Categorical (y/n)
	Cattle feed	Are cattle fed cereals or concentrates	Categorical (y/n)
	Feed accessibility	Is palatable feed (cereals or concentrates) accessible to badgers (in the farmers view over a 12 month period)	Categorical (never / sometimes / all year)
	Cattle sheds	Number of cattle sheds (buildings regularly housing cattle)	Categorical (0–2, 3–4, ≥5)
	Max cattle capacity	Maximum number of cattle housed at any time on the farm	Continuous
	Dairy	Presence or absence of dairy cattle on farm	Categorical (y/n)
	Lights	Presence of outdoor lighting which is on at night	Categorical (y/n)
	House	Presence of a house / dwelling present on farm	Categorical (y/n)
	Dogs	Presence of dogs on the farm	Categorical (y/n)
Other variables	Season	Period of time when camera surveillance carried out	Categorical (spring: Apr-May, summer: June—July, autumn: Aug—Sep)
	Worm nights	Measure of rainfall. Number of nights with ≥2mm of rain in previous 72 hours, based on Kruuk and Parish [29].	Continuous

### 2.3 Statistical analyses

#### 2.3.1 Factors effecting badger visits

In order to investigate factors related to badger visits we conducted a series of generalized linear models using the R package ‘lme4’ [30]. We carried out one set of analyses to investigate factors influencing the presence /absence of badger visits at farms (badger presence = ≥1 badger observations over the period), with a binomial (0/1) response. We then conducted a second set of analyses to investigate factors influencing the frequency of badger visits (proportion of survey nights on which badger visits were photographed) at the subset of farms visited by badgers, using a binomial (proportion) response. In both analyses, fixed effects included in the models consisted of 14 variables outlined in Table 1. Although the number of cameras deployed varied among farms, camera number was not included as a measure of survey effort in the analyses. This is because the number of cameras was dictated by the size of the farm and number of access points. As such, larger farms (with more cameras) could not be viewed as necessarily having higher survey effort than smaller farms (with fewer cameras).

Models containing all first order combinations of variables (Table 1) were evaluated via model averaging using the ‘MuMIn’ package (version 1.15.6) [31, 32]. Prior to analyses all continuous predictor variables were standardised to mean = 0 and sd = 0.5 following [32], such that effect sizes (model coefficients) were on comparable scales. Average coefficients were calculated from a top model selected using a corrected Akaike Information Criterion (adjusted for small sample size ΔAICc) cut-off of six units, which has been suggested as having a 95% probability of containing the most parsimonious model [33]. Model ranking and averaging of proportion models used QAIC followed Bolker [34], as proportion data (badger visit rate) had a skewed distribution and initial analyses indicated over dispersion. In both analyses, variable coefficients with 95% confidence intervals that did not span zero were deemed to have a consistent or ‘significant’ positive/negative effect on badger visits [32].

Lowess plots and descriptive statistics were used to investigate the functional form of continuous predictor variables and to assess whether linearity assumptions were met (and whether transformations were required). Collinearity among predictors was investigated using Pearson’s correlations and by calculating variance inflation factors for final models. Model fit, heteroskedasticity, influential points and leverage values were investigated using Pearson residuals and a range of plots and functions using the ‘car’ package in R, following [35]. Spatial autocorrelation was investigated by calculating Moran’s I for both response and predictor variables.

#### 2.3.2 Assessing the accuracy of model predictions

Once final models comprising only variables with consistent positive/negative effects had been identified for badger presence/absence and badger visit rates, the predictive accuracy of these models was tested. Prediction accuracy was tested using the 155 farm ‘training data set’, and a ‘test data set’ of 40 farms not used in the earlier analyses. The test data set farms had been monitored using trail cameras for a continuous 12 month period as part of an earlier, independent study investigating the effectiveness of biosecurity measures in preventing badger visits [14]. These farms were located in Gloucestershire and were randomly selected, although all farms had at least 30 cattle housed for at least part of the year (see [14] for details). Only footage prior to the application of biosecurity measures at these farms was used.

Models of presence/absence produce a predicted probability of 0 to 1 for each farm. To change this into a categorical prediction (i.e. yes or no) required setting a cut-off or threshold value (above which badgers were classed as present and below which they were considered absent) [36]. We therefore calculated receiver operating characteristic (ROC) curves, which plot sensitivity (the probability that farms with badger visits are correctly identified) against 1—specificity (the probability that farms with no badger visits are correctly identified) for all potential cut-off values between 0 and 1. Model accuracy was calculated using the area under the curve (AUC), which acts as a single summary statistic of diagnostic accuracy [37]. Models with AUC values of 0.5 are viewed as uninformative (i.e. no better than a random guess) while values of 1 indicate a perfect test with 100% accuracy. Swets [38] suggests that values of 0.5<AUC≤0.7 = low accuracy, 0.7<AUC≤0.9 = moderate accuracy (and ‘useful for some purposes’) and AUC>0.9 = high accuracy. We also chose a single cut-off value that maximised model accuracy (i.e. % of farms correctly classified) in order to produce presence/absence predictions for individual farms. Changing this cut off could increase sensitivity or specificity, depending on whether the priority was to identify farms with visits (i.e. to make sure that measures are applied where they are needed), or to minimise false positives (i.e. to avoid applying measures at farms where they are not needed). This is discussed at greater length later in the discussion. Agreement between predictions and observed badger presence/absence was calculated using Cohen’s Kappa statistic, and by the construction of ‘confusion matrices’. Finally, the accuracy of badger rate predictions were investigated using correlation analyses to compare observed and predicted values, for both the 155 farm training data set and the 40 farm test data set.

#### 2.3.3 Creation of an interactive farm assessment tool

In order to calculate and display our predictions of the likelihood of badger visits to unsurveyed farms, we created an interactive app BFAT or ‘Badger Farm Assessment Tool’ using the R package shiny [39]. The shiny app consists of an interactive interface, whereby users can enter their farm characteristics in relation to variables identified as important predictors of badger visits. These parameters are then used to produce an individual farm score for badger visits, displayed via several figures. A range of methods are available for visualising and communicating probability and risk, with several studies demonstrating that there is significant variation among people with regard to what formats they prefer or best understand [40, 41]. Individual farm scores were therefore displayed using a range of formats, including text and pictographs.

## Results

**Fig 2:**
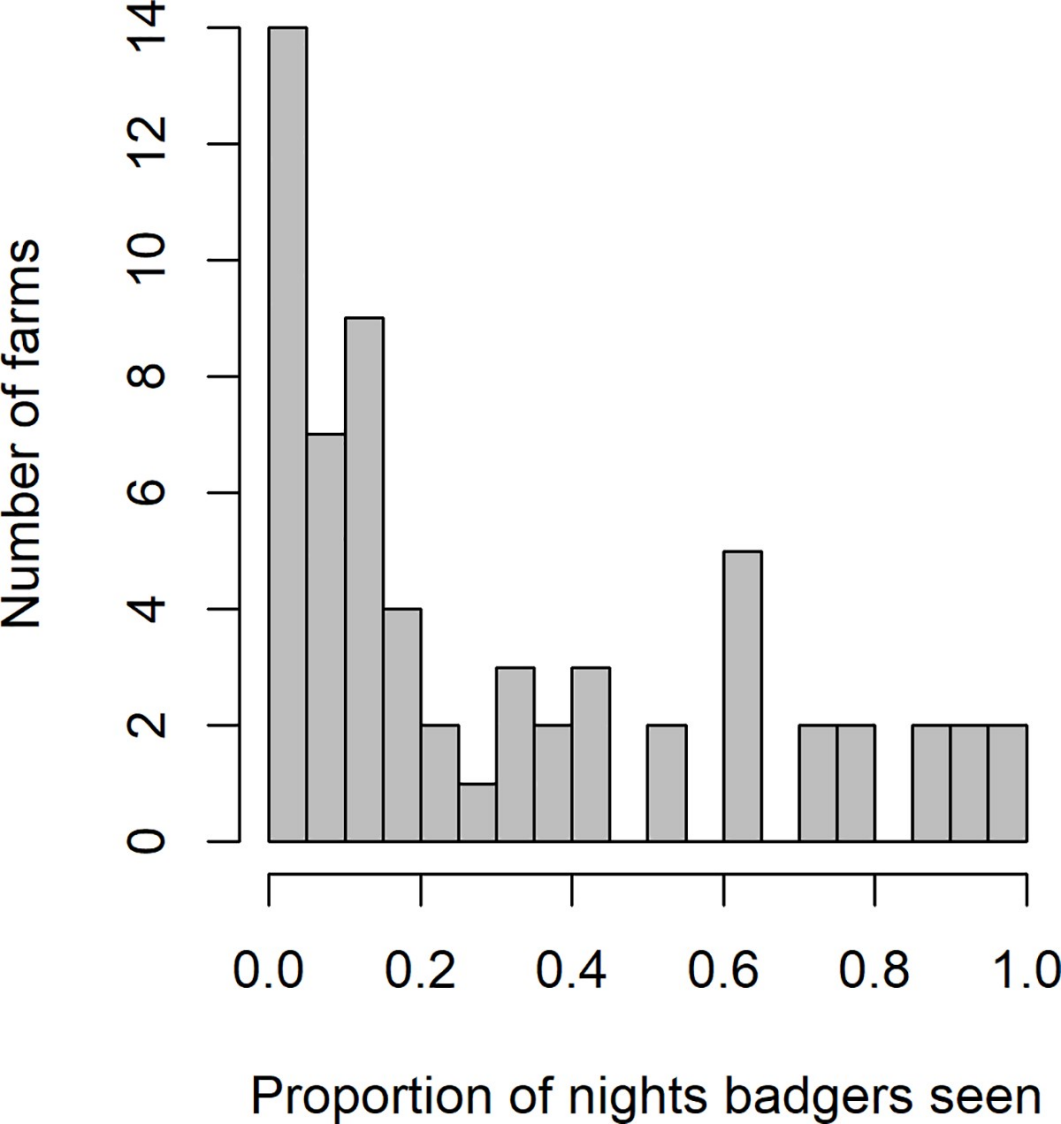
Histogram displaying the proportion of survey nights when badger visits were recorded on 62 farms (training data) where badger visits occurred.

A total of 155 farms were included in the study, consisting of 82 beef farms, 42 dairy, 19 mixed (beef and dairy), 11 calf rearing/suckler herds and one rare breed herd (beef). Badgers were recorded on camera at 40% (62/155) of farms monitored. Field surveys recorded badger setts within 500 m (of the centre of the farm building complex) at 133/155 farms (86%), and active badger setts at 120/155 farms (77%). The proportion of camera nights where badgers were observed in farmyards varied markedly amongst farms (Fig 2), with badgers observed on more than 50% of camera nights at 11% (17/155). Badgers were most often recorded in cattle housing and yards, followed closely by feed stores, feed and/or water troughs and hay/straw barns, and least often at silage clamps and calf pens (Table 2), although this variation in visits was not significant (*X*^2^_6_ = 6.778, p = 0.34). Of the 155 farms, 44 were under TB restrictions at the time of the study. In univariate analyses the TB status at the farm was not associated with the presence of badgers on camera (null model AIC = 184.94, badger presence AIC = 182.07, Deviance_1_ = 2.86, p = 0.09) or with the proportion of survey nights where badgers were seen (at farms where badgers were present, null model AIC = 65.68, proportion nights badgers seen, AIC = 67.44, Deviance_1_ = 0.23, p = 0.62).

**Table 2:** Badger visits recorded on camera to different areas within the surveyed farms.

Area of Farm	Number farms location monitored	% Farms where badgers seen
Cattle housing	144	27.1
Yard	140	27.1
Feed store	99	26.3
Feed trough	55	25.5
Hay/Straw barn	111	25.2
Silage clamp	45	13.3
Calf pen	33	12.1

There was evidence of spatial autocorrelation among farms in relation to the presence of badgers in farmyards (Moran’s I observed = 0.05, expected = -0.006, p = 0.005), but not in the frequency of badger visits (Moran’s I observed = -0.005, expected = -0.006, p = 0.95). However, there was also spatial autocorrelation in the badger sett density variable (observed = 0.17, expected = -0.006, p = <0.005) and distance to nearest sett variable (observed = 0.02, expected = -0.006, p = <0.005), suggesting badger activity is clustered in the landscape. Spatial autocorrelation was not controlled for in subsequent analyses, as not to dilute or mask the effects of these badger abundance variables, which are likely to be key factors influencing badger visits.

### 3.1 Factors effecting presence / absence of badger visits

**Fig 3:**
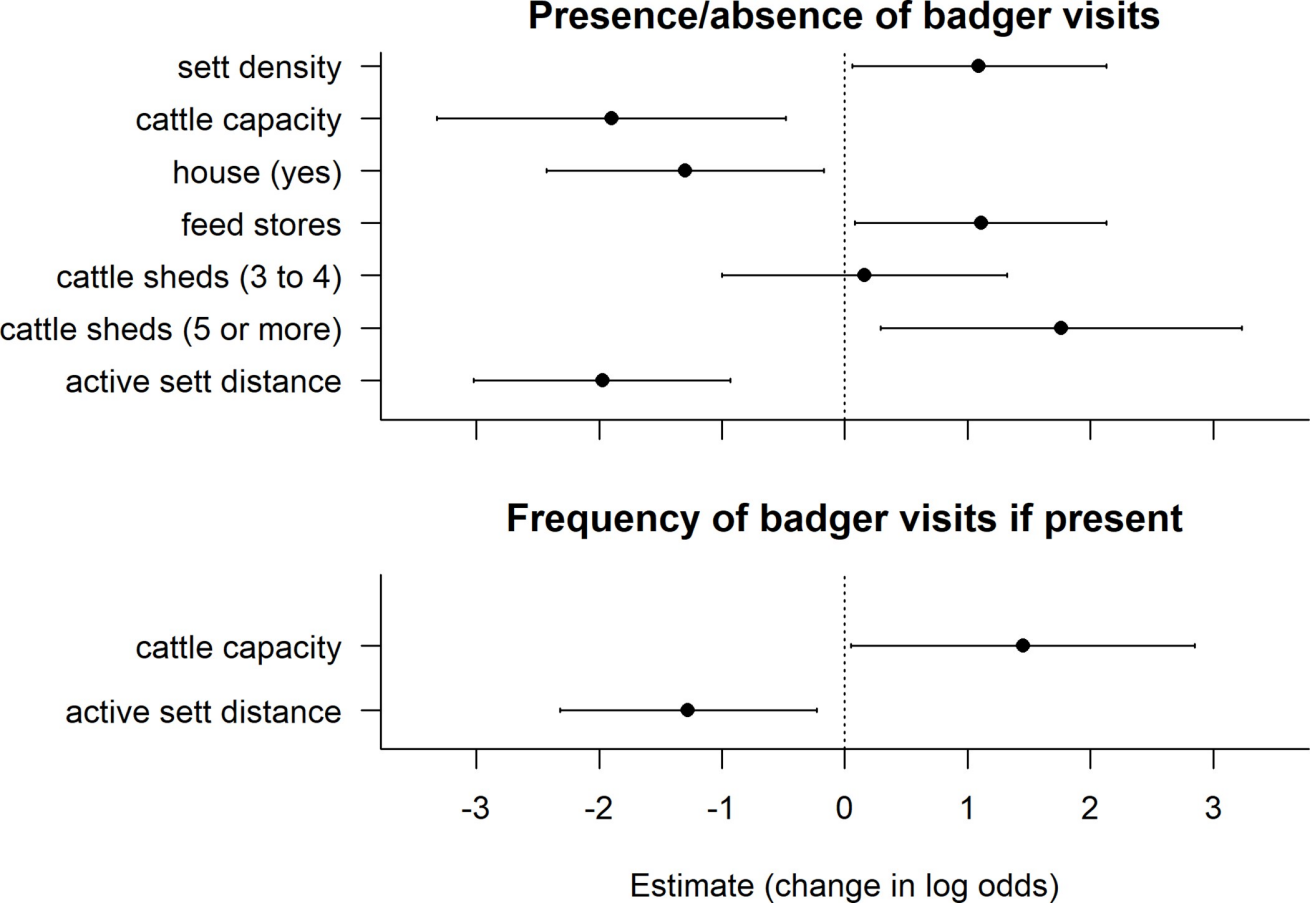
Factors affecting the likelihood of badger visits (top) and the frequency of badger visits at farms where they were present (proportion of nights badgers seen, bottom). Values are average model coefficients (change in log odds) calculated for variables included in the top model set (≤ 6 AIC, Table A in S1 Appendix). Arrows indicate 95% confidence intervals. Model-averaged regression slopes are considered important if they are consistently positive or negative (i.e. their confidence intervals do not span zero). Continuous variables (sett density, cattle capacity, active sett distance and feed stores) were standardised (mean = 0, sd = 0.5) prior to analysis.

**Fig 4:**
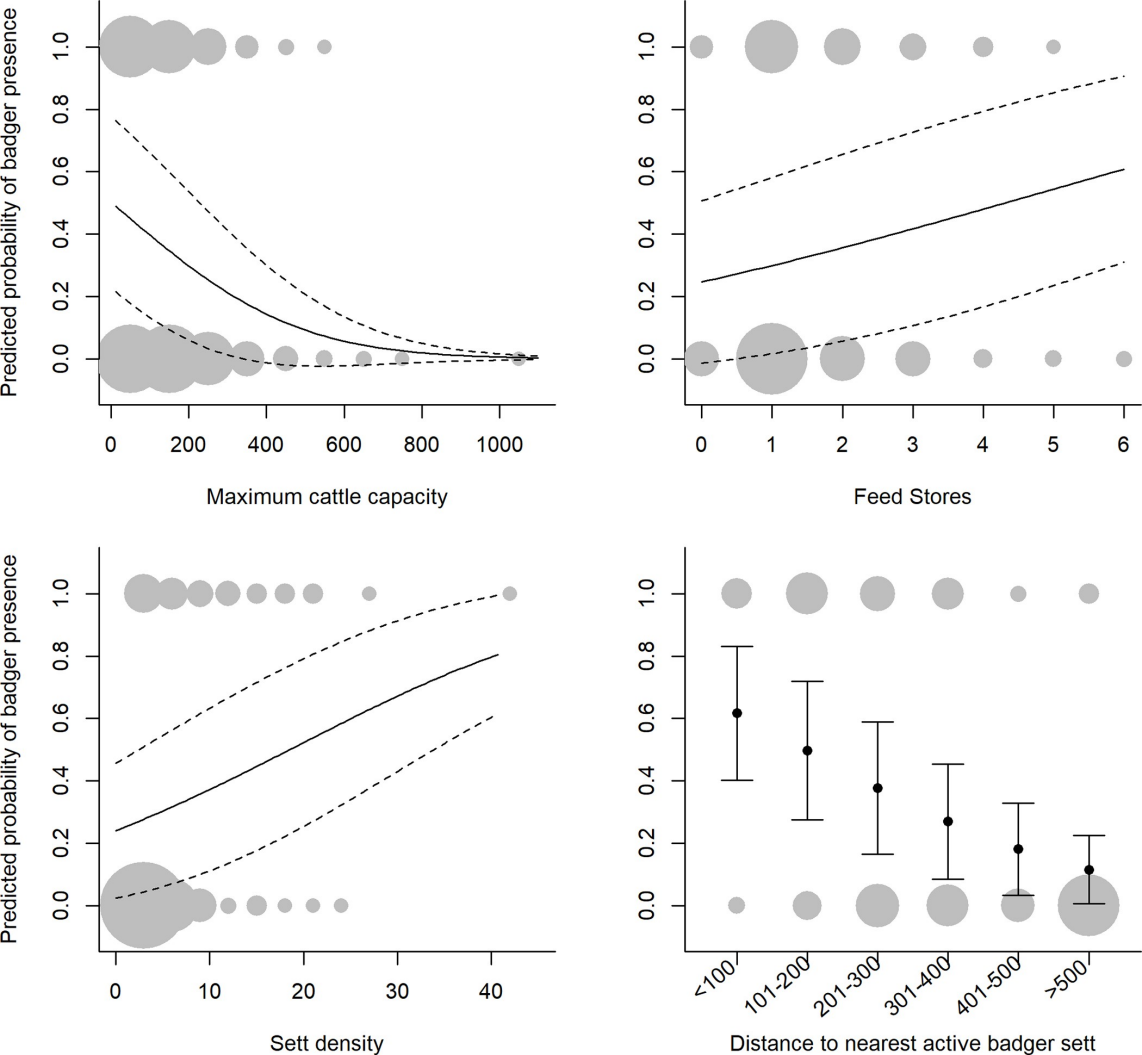
Predicted probability of badgers being present (at least one observation on camera) in farmyards in relation to four farm level variables; cattle capacity, feed stores, badger sett density and distance to nearest active badger sett. Bold lines represent the marginal predicted probability and dashed lines (or error bars) the standard deviation. Circles summarise the raw data, with the size of the grey point scaled to the number of observations in that group (smallest point = 1, largest point = 45).

The likelihood of cameras recording badgers on farms was significantly related to the presence of badger field signs within a 500 m radius, and to several farm characteristics (Figs 3 & 4, full model coefficients are in Table A S1 Appendix). Likelihood increased with the density of badger setts surrounding the farm and with proximity to the nearest active sett, such that farms with closer setts and more setts were more likely to be visited (Figs 3 & 4). Sett density had a skewed distribution with >30 setts per km^2^ recorded at one farm (11 setts recorded, despite only 34% of the 500m radius being accessible), though removing this outlier had no effect on the coefficient for this variable (outlier included: coef = 1.09, CI = 0.06–2.13. outlier removed: coef = 1.05, CI = 0.03–2.12). Badgers were more likely to be present at farms with higher numbers of feed stores, and with more cattle sheds, particularly where there were more than five cattle sheds on the farm (Fig 3). Badgers were less likely to be present in farmyards where there was a house or dwelling than in those without (Fig 3). The likelihood of badger visits also declined with the cattle capacity (max number of cattle that could be housed), such that farms with larger herds were less likely to be visited (Figs 3 & 4). Cattle capacity had a skewed distribution, with high values of ≥600 at four farms. Cattle capacity still had a consistent negative effect with these outliers excluded, although the coefficient was lower (-1.60 compared to -1.90 if included) with confidence intervals closer to zero (-3.11 to -0.01, compared to -3.32 to -0.48). Several other variables were contained in top models (≤6 AIC), but the estimates spanned zero, suggesting an inconsistent or non-significant effect (Table B in S1 Appendix). The overall top model set (≤6 AIC) was very large and consisted of 150 candidate models. However, the coefficients were very similar to a smaller top model set using a cut off of ≤2 AIC (n = 8 models, Table B in S1 Appendix). Model diagnostics indicated no significant issues with outliers (high leverage values), heteroscedasticity or collinearity among variables. Hosmer-lemeshow tests indicated no evidence of poor model fit for the top model set (*X*^2^_5_ = 5.53, p = 0.35).

### 3.2 Factors affecting the frequency of badger visits

**Fig 5:**
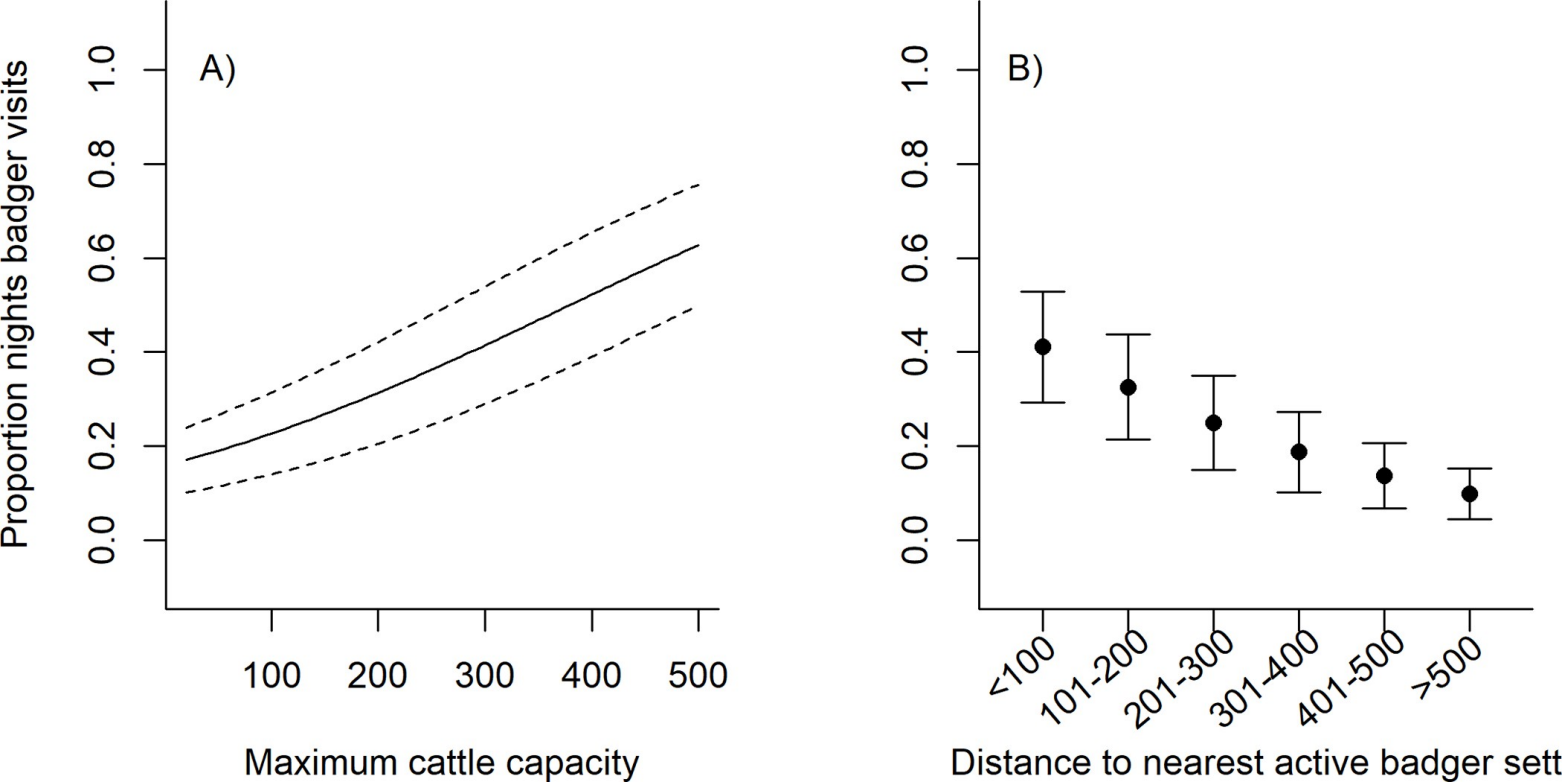
Predicted badger visitation rate (proportion of nights badgers observed) in relation to the maximum cattle capacity on the farm and the distance to the nearest active badger sett. Bold lines represent the marginal predicted probability and dashed lines (or error bars) the standard deviation.

At the 62 farms visited by badgers, the frequency of visits (proportion of nights where visits were recorded) was related to the cattle capacity at the farm and the distance to the nearest active badger sett (Figs 3 & 5). As with badger presence/absence, the frequency of badger visits was negatively related to the distance to the nearest active sett, such that farms with closer active setts had a higher number of visits (Fig 5). However, in contrast to the presence/absence analysis the cattle capacity (max cattle housed) was positively related to badger visits, such that farms with higher cattle capacity had a higher frequency of visits (Fig 5). As with the badger presence/absence analyses the overall top model set (≤6 AICc) was very large and consisted of 392 candidate models. However, the coefficients were very similar to a smaller top model set using a cut off of ≤2 AICc (n = 19 models, Table B in S1 Appendix).

### 3.3 Accuracy of model predictions–badger presence/absence

**Fig 6:**
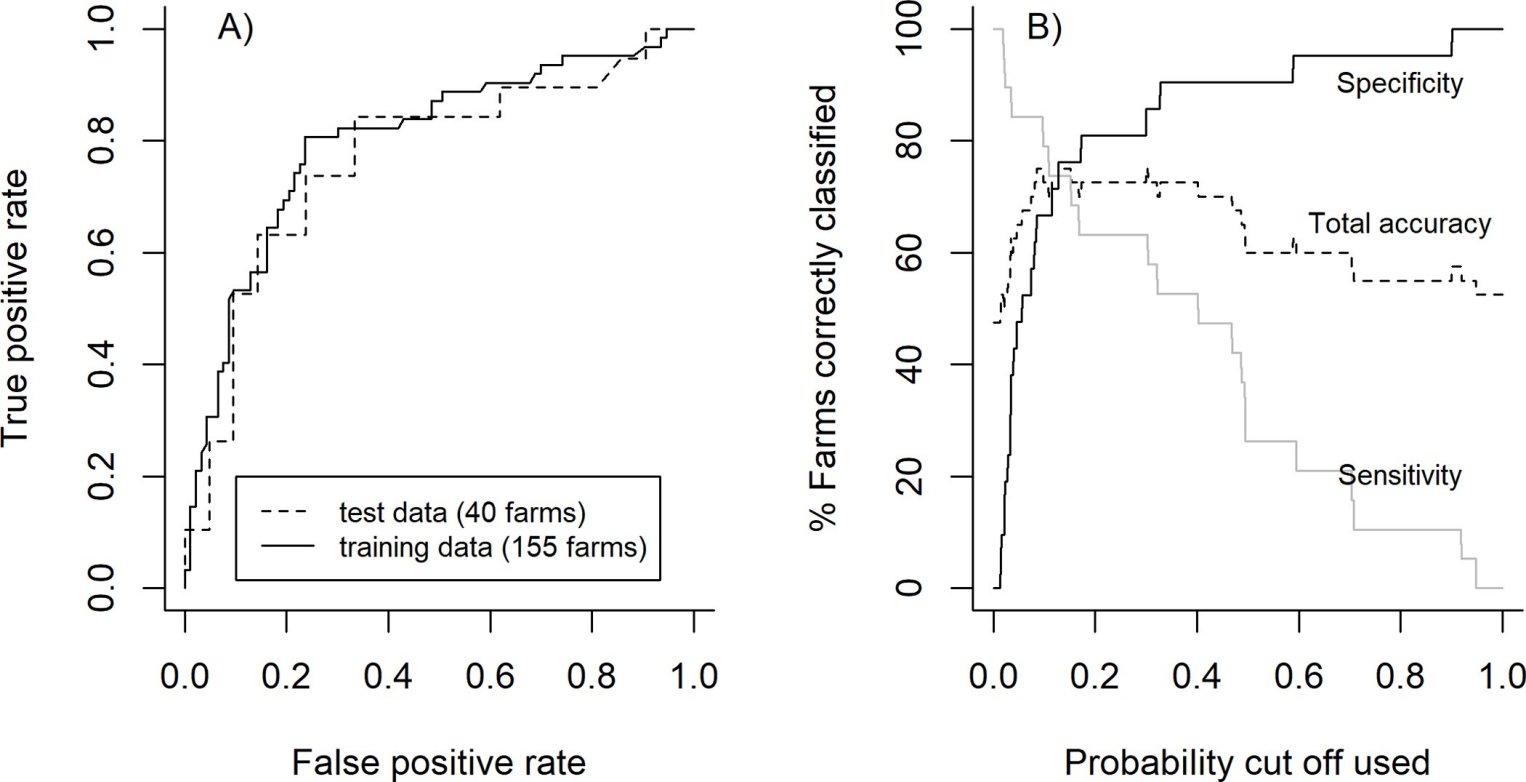
Accuracy of model at predicting badger presence/absence at 40 farmyards used as a test data set. Figure A is the ROC (receiver operator curve), which displays the true positive rate (proportion of farms with badgers present identified) vs the false positive rate (proportion of farms wrongly classified as having badgers present) for a varying cut off value. Figure B displays the percentage of farms correctly identified as having badgers present (sensitivity—grey line), badgers absent (specificity—black line) and total accuracy (dashed line), relative to the cut off used (farms with a predicted probability above this value are classed as having badgers present).

Six variables were found to be related to the likelihood of badgers being recorded in farmyards (Fig 3). When applied to the 155 farm training data set used in the analyses, the averaged variable coefficients for the badger presence model (using the average coefficients in Table 1) had an ROC AUC of 0.80 (95% CI = 0.73–0.88, Fig 6). When applied to the 40 test farms (not used in the analyses) the ROC AUC had a similar value of 0.77 (95% CI = 0.61–0.92, Fig 6), suggesting that the model was ‘moderately accurate’ [38]. Maximum accuracy (% farms correctly classified) with the 155 farm training data set was 78.0% using a cut-off value of 0.261–0.271 (midpoint = 0.265) to classify farms as having badgers present. Applying a cut-off value of 0.265 to the 40 test farms correctly identified 12/19 farms with badgers present (sensitivity = 63.2%), 17/21 farms with badgers absent (specificity = 81.0%) and 29/40 farms overall (total accuracy = 72.5%, Table 3). Using this cut-off the positive predictive value (PPV: % of farms where badgers were predicted as present where badgers were observed) and negative predictive value (NPV: % of farms where badgers were predicted as absent where badgers were not observed) were 70.8% and 75.0% respectively (Table 3). Adjusting the cut-off could increase sensitivity or specificity, as well as PPV and NPV, although this would compromise overall accuracy (Fig 6). It was also possible to increase overall accuracy to 75% using the 40 test farms and a lower cut-off (0.128 to 0.152, Fig 6), although this significantly reduced accuracy (to 64.5%) when applied to the 155 training farms.

**Table 3:** Confusion matrix displaying model predictions for badger presence/absence in farmyards (using a cut-off of 0.25) compared to observed survey results (based on camera sightings) at the 40 test farms where badger activity was monitored for 12 months. Values for sensitivity (% of farms with badgers present correctly identified) and specificity (% of farms with badgers absent correctly identified), PPV (positive predictive value: % of farms where badgers were predicted as present where badgers were observed) and NPV (negative predictive value: % of farms where badgers were predicted as absent where badgers were not observed).

		Observed		
		Absent	Present	
Model Prediction	Absent	17	7	PPV = 70.8%
	Present	4	12	NPV = 75.0%
		Specificity = 81.0%	Sensitivity = 63.2%	

### 3.4 Accuracy of model predictions–badger visit rate

**Fig 7:**
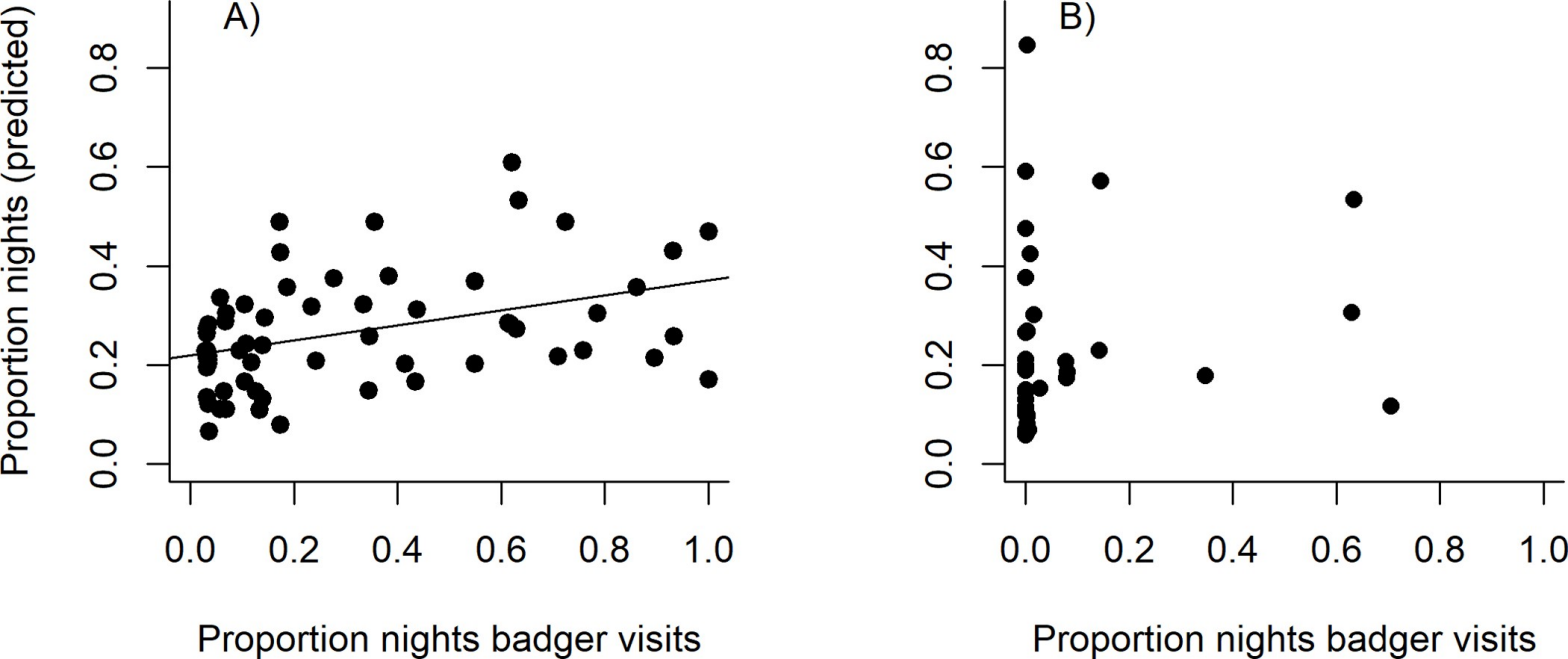
Observed rate of badger visits to farmyards (proportion of nights of observation when visits took place) compared to the predicted rate (based on model parameters, Figs 3 and 5) at the 62 training farms analysed to produce the model (Fig A) and at the 40 test farms (Fig B).

Two variables were found to be related to the badger visit rate at farms (Figs 3 and 5). The predicted rate was significantly correlated with the observed rate at the 62 training farms where badgers were present (*t*_*60*_ = 3.34, p = 0.001, r = 0.40 Fig 7). However, when applied to the 40 farm test data set, the predicted visit rate showed only a poor correlation with the observed rate, either at all 40 farms (*t*_*38*_ = 0.97, p = 0.34, r = 0.16, Fig 7), at the farms where badgers were present (at least one camera sighting, *t*_*17*_ = 0.34, p = 0.74, r = 0.08, Fig 7) or at farms which were predicted as having badgers present (using the presence/absence model, *t*_*14*_ = 0.30, p = 0.77, r = 0.08). However, among test farms where badgers were present (at least one visit), those that were predicted as having badgers present (based on the presence/absence model) had higher maximum monthly visit rates (i.e. the maximum visit rate recorded in a given month over the 1 year survey period) than those which were predicted as having badgers absent (coefficient = 0.41, *X*_*1*_^*2*^ = 4.78, p = 0.03). This suggests that the presence/absence model is more likely to identify farms with higher rates of visits than those with low rates.

### 3.5 Communicating farm level risk scores

**Fig 8:**
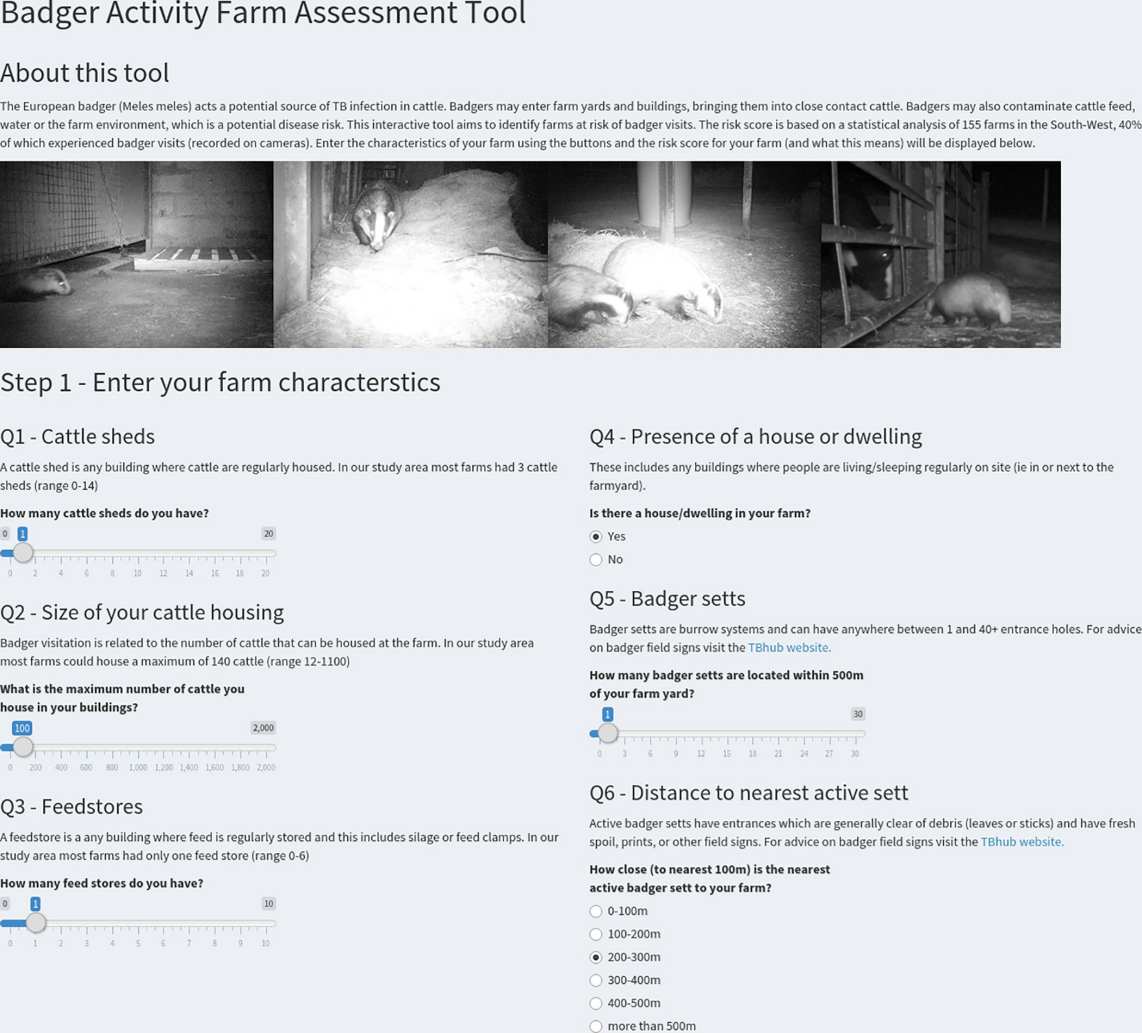
Interactive tool displaying an interface for entering farm characteristics.

**Fig 9:**
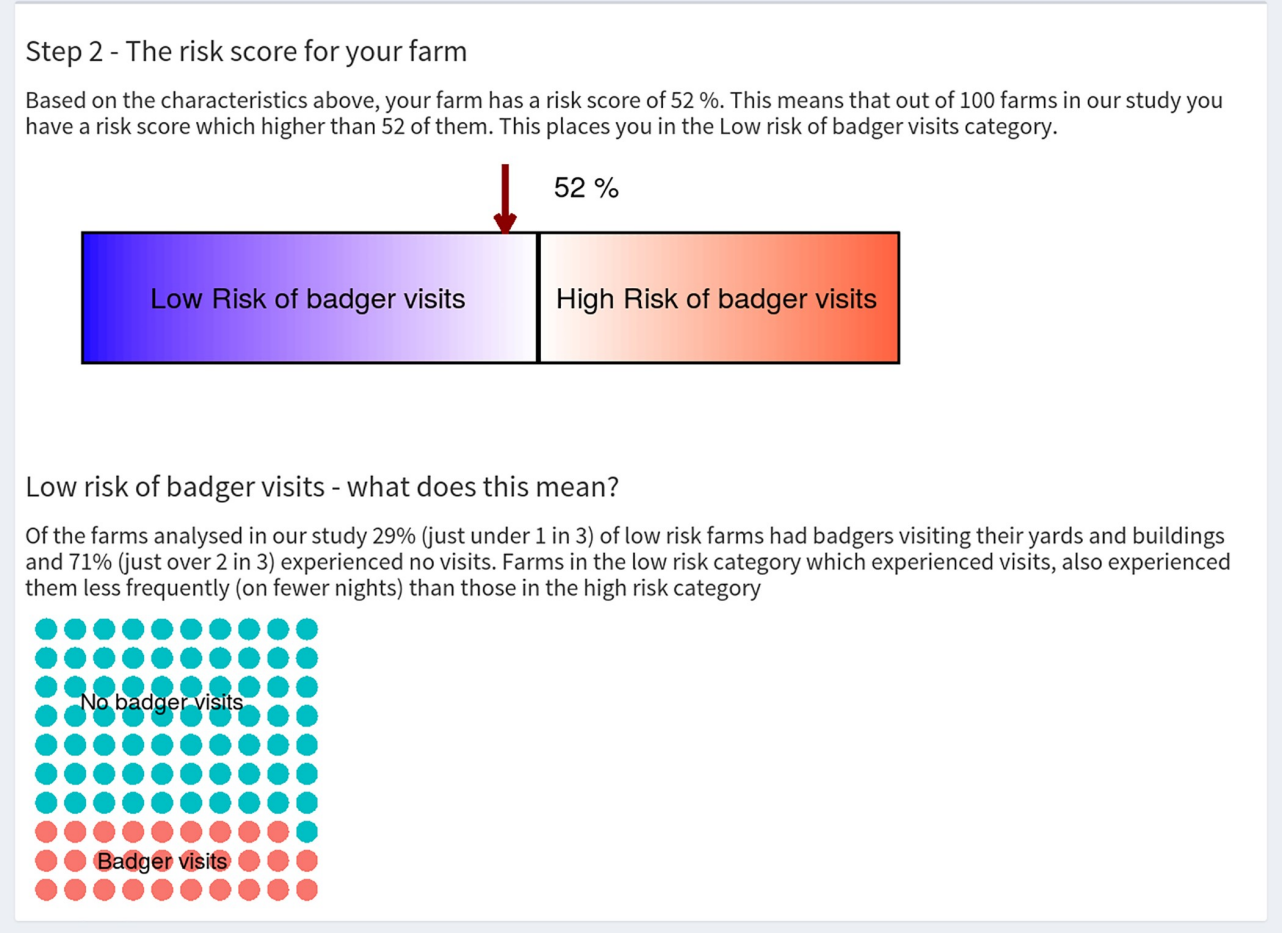
Example output from the interactive tool displaying the percentage risk score relative to other farms and an illustration of what this means using a 10X10 grid.

In order to communicate farm level risk scores to stakeholders we developed an interactive tool using shiny in R https://btb-statistics.shinyapps.io/badger_farm_assessment_tool_prototype/ (code for the app is in S1 Appendix). Users can enter data on the six farm-level variables that were significantly related to badger presence/absence in the analyses in this study (Fig 8). The tool then uses the coefficients (Fig 3, Table A in S1 Appendix) to predict the probability of badger visits to yards or buildings taking place on that farm. Rather than display a single probability value, the predicted value is given as a percentage (quantile) compared to the predicted probability values for the 195 farms in this study (155 training farms + 40 test farms). The relative percentage risk score is divided into ‘low likelihood of badger visits’ and ‘high likelihood of badger visits’ using a cut-off of 0.25 probability, which equates to the 54^th^ percentile in the 195 farms (Fig 9). This risk score is then communicated using a sliding scale or ‘thermometer’ style pictograph, and is described in the text. An explanation of the low or high risk category is displayed below this using a 10 x 10 grid (with each point representing a farm), which is a standard approach in risk communication [40]. The numbers on the grid reflect the positive predictive values displayed in Table 3. Links are provided to government approved biosecurity advice at the bottom of the tool.

## Discussion

Badgers were observed in 40% of the 155 farmyards monitored over the one month survey period, suggesting that a significant proportion of farms in the survey area experienced some level of badger activity. The frequency of badger visits (proportion of nights where badgers were seen) among the farms where badgers were present was highly variable. As in Judge et al. [14], a minority of farms (approx. 10%) were frequently visited by badgers with others receiving comparatively low levels of activity. Given that monitoring only took place for one month, and that surveillance coverage was not complete, this is likely to be an underestimate of levels of visits to the sampled farms. However, it is also possible that the farms in this study were not representative of those in the wider landscape, as farms were not randomly selected. For example, farmers concerned about badger activity may have been more likely to take part in the study than those which were not concerned about badgers. The sample size in this study was large (n = 155) and included a mix of farms, but the non-random selection is an important limitation, such that the results here may not be directly applicable to all farms in the wider landscape. Nevertheless, our results are similar to those from previous intensive monitoring of 40 randomly selected farms in Gloucestershire which formed part of our test data) and further highlights that farmyards could provide opportunities for transmission of infection between badgers and cattle [6, 12].

We found that the presence or absence of badgers and the frequency of their visits was related to several farm characteristics, including measures of badger abundance. Unsurprisingly, an active sett in close proximity increased the likelihood that badgers would be observed in farmyards and increased the frequency of badger visits. The density of badger setts (a potential proxy for badger density [42]) within 500 m of the farm was also related to badger presence, but not to the frequency of visits. This contrasts slightly with results from [17] who found a correlation between badger density and farm visit rate. Badgers exhibit variable foraging strategies [43] with higher individual variation associated with competition for resources [44]. Competition at higher densities may, therefore, favour the utilisation of resources found in farmyards. High population density may also increase the likelihood of risky or bold behaviour. For example, studies have shown that badgers in small groups (<4 animals and those with low levels of activity) are more wary, or ‘neophobic’ [45] and are less likely to exploit novel anthropogenic resources [46].

Previous observational and tracking studies suggest that badger activity in farmyards is largely associated with foraging behaviour, with frequent observations of visits to feed stores and consumption of stored cattle feed [10, 11]. The number of feed stores or cattle sheds in the current study may be an indication of the number of potential feeding opportunities within the farm, which may explain why these characteristics were positively related to the presence of badgers in farmyards. The likelihood of badgers being present was also related to the number of cattle that could be housed on the farm (cattle capacity) and the presence of an occupied house or dwelling. Badgers were less likely to visit farms with higher cattle capacity and with the presence of a house or dwelling. It is not clear why this should be, but one possible explanation is that both variables are indicative of higher levels of human activity on the farm, which could deter badger visits. Interestingly, although the presence of badgers was negatively related to the cattle capacity, where badgers were present the frequency of their visits was positively related to this variable. This could be because although badgers may be generally disinclined to visit larger, busier farms, where they are present (typically on smaller farms with lower capacity) the number of animals housed is a proxy for increasing quantities of feed and hence more opportunities for foraging.

The aim of the present study was not only to identify ecological factors associated with badger visits to farmyards, but to test whether these variables could be used as a predictive tool. The capacity to identify those farms most likely to experience badger visits could be used to target advice to farmers and hence to direct the deployment of biosecurity measures. Our models could predict badger presence with 73% accuracy, with overall sensitivity and specificity of 63% and 81% respectively (using a probability cut-off of 0.265). Using a different cut off value could increase specificity or sensitivity, which could be desirable under certain circumstances. For example, if the disease risk in the local badger population was perceived to be low, or if measures were likely to be very expensive a high sensitivity (to avoid placing measures where they are not needed) could be advantageous.

Using a cut-off of 0.25, farms identified by the model as having a high likelihood of badgers being present typically also had a higher frequency of badger visits than those not identified. However, overall the model of badger visit frequency, as opposed to presence/absence, had poor predictive power, and was unable to distinguish between farms with high or low levels of activity. The extent to which wild animals exploit anthropogenic food sources and environments is likely to be influenced by a variety of behavioural factors, including associations with other individuals with a tendency to use these resources [47], or could be mediated through learning from parents [48]. Badger visits to farmyards could also be influenced by such processes, or by chance events which makes the behaviour difficult to predict from ecological factors alone.

Previous studies have highlighted that badgers entering farmyards may present potential opportunities for disease transmission to cattle, although the mechanisms and the magnitude of the risks are unclear [6, 12, 49]. In addition, the absolute disease risk from any given badger is likely to depend on the level of infectiousness, which can vary significantly amongst individuals and social groups [50]. Transmission could occur from chance events, which may depend on the nature of the badger behaviour in farmyards, such as how their activity patterns overlap directly or indirectly with cattle. Such events would need to result in sufficient exposure to *M*. *bovis*, although the minimum infectious dose for cattle is low, at 6–10 bacilli [51]. Intuitively farms with a higher frequency of badger visits should have a higher risk of infection, although we found no evidence that badger visits was associated with the current TB status of the farm (in simple univariate analyses). This is not that surprising, as transmission may require a particular set of circumstances, such that any relationship between the two may be weak, highly variable and/or non-linear in nature. Several studies have also demonstrated that TB risk is influenced by a complex mix of factors including badger activity, as well as cattle movements and other farming practices [52]. Quantifying the risk from badgers farm visits is therefore likely to require large multivariate analyses, considering a range of factors.

Although the model for predicting the likely presence of badgers in farmyards did not have perfect predictive accuracy, these initial findings nonetheless have potential implications for the targeting of biosecurity measures [14]. Often studies of wildlife management problems describe ecological patterns or relationships, but fail to identify how such knowledge can be effectively transferred to stakeholders in order to inform their choices. Communicating risk is notoriously difficult, although numerous examples exist particularly in the field of human medicine [40], as well as other disciplines. In an attempt to address this challenge we created an interactive web based tool. Based on the models in this study the tool allows users to enter their farm details and produce a relative risk of the likelihood of badger visits. Variables relating to the farm buildings or herd size will be straight forward to enter and most farmers will have an approximate idea of the number and location of setts on their land, although on average these numbers tend to be slight underestimates [53]. Farms predicted as being at high risk of receiving visits could experience a range of badger activity levels, which could in turn relate to highly variable levels of disease risk. In addition, each farm will pose its own challenges and there may be other factors or priorities which influence the application of biosecurity measures [14]. It is envisaged that the output from the assessment tool could therefore form part of a wider discussion between farmers and vets in developing a biosecurity plan for a farm. Such a discussion could consider local TB risk in their area, the potential costs of badger-proofing farmyards, and other information on disease patterns or risk from government or private animal health professionals.

In summary, we found that badger visits to farm buildings occurred at a significant proportion of farms at the landscape scale and that the presence of badgers could be predicted on the basis of farm characteristics. An interactive ‘farm assessment tool’ developed using the model outputs, represents a first step in providing stakeholders with information to help them target interventions aimed at reducing opportunities for TB transmission amongst badgers and cattle. Collection of further data, along with future research into ecological and behavioural factors which influence badger activity in farmyards may help to improve the predictive ability of these models. A similar approach to that described here could also be applied to identify areas of risk in other situations, such as pasture fields, in order to direct biosecurity measures in the wider farming environment.

## Supporting information

S1 Appendix Table A–Averaged model coefficients from top model set (≤6 AICc) investigating the likelihood of badger presence/absence at surveyed farms (n = 155). Variables in bold are those with 95% confidence intervals that did not span zero (for log odds coefficients), or one (for odds ratios, OR). Variable relative importance and the total number of models in the top set containing the variable (‘n models’), are also displayed. Table B–Comparison between coefficients generated using a top model set of ≤6 AICc and ≤2 AICc. From models investigating badger presence/absence and badger visitation rate. Only variables with 95% confidence intervals that did not span zero are displayed. Number of models in top model set of badger presence/absence is 93 for ≤6 AICc and 6 for ≤2 AICc. Number of models in top model set of badger visitation rate is 328 for ≤6 AICc and 20 for ≤2 AICc.
(DOCX)

S2 Appendix R code for Shiny app. (DOCX)

S3 Appendix Spreadsheet containing data for analyses. (DOCX)
