# Supplementary material for: Predicting badger visits to farm yards and making predictions available to farmers
Source: PLoS One. 2019 May 24;14(5):e0216953. doi: 10.1371/journal.pone.0216953 (PMC6534311; doi:10.1371/journal.pone.0216953)
Supplement: S1 Appendix — Table A–Averaged model coefficients from top model set (≤6 AICc) investigating the likelihood of badger presence/absence at surveyed farms (n = 155). Variables in bold are those with 95% confidence intervals that did not span zero (for log odds coefficients), or one (for odds ratios, OR). Variable relative importance and the total number of models in the top set containing the variable (‘n models’), are also displayed. Table B–Comparison between coefficients generated using a top model set of ≤6 AICc and ≤2 AICc. From models investigating badger presence/absence and badger visitation rate. Only variables with 95% confidence intervals that did not span zero are displayed. Number of models in top model set of badger presence/absence is 93 for ≤6 AICc and 6 for ≤2 AICc. Number of models in top model set of badger visitation rate is 328 for ≤6 AICc and 20 for ≤2 AICc. (DOCX) [file pone.0216953.s001.docx]

**S1 Appendix**

**Table A – Averaged model coefficients from top model set (≤6 AICc) investigating the likelihood of badger presence/absence at surveyed farms (n=155). Variables in bold are those with 95% confidence intervals that did not span zero (for log odds coefficients), or one (for odds ratios, OR). Variable relative importance and the total number of models in the top set containing the variable (‘n models’), are also displayed.**

| Variable | Coefficient | coef L95% | coef U95% | OR | OR L95% | OR U95% | Relative importance | n models |
| --- | --- | --- | --- | --- | --- | --- | --- | --- |
| (Intercept) | -0.10 | -1.28 | 1.08 | 0.90 | 0.28 | 2.93 | - | - |
| **Distance to nearest active badger sett*** | **-1.97** | **-3.02** | **-0.93** | **0.14** | **0.05** | **0.39** | **1.00** | **150** |
| **Cattle sheds (5 or more)** | **1.76** | **0.29** | **3.23** | **5.81** | **1.34** | **25.26** | **0.93** | **131** |
| Cattle sheds (3 to 4) | 0.16 | -1.00 | 1.32 | 1.17 | 0.37 | 3.73 | 0.93 | 131 |
| **Feed stores*** | **1.11** | **0.08** | **2.13** | **3.02** | **1.08** | **8.44** | **0.84** | **115** |
| **House (yes)** | **-1.30** | **-2.43** | **-0.17** | **0.27** | **0.09** | **0.84** | **0.93** | **133** |
| **Max cattle capacity*** | **-1.90** | **-3.32** | **-0.48** | **0.15** | **0.04** | **0.62** | **1.00** | **150** |
| **Badger sett density*** | **1.09** | **0.06** | **2.13** | **2.98** | **1.06** | **8.38** | **0.86** | **117** |
| Lights (yes) | 0.71 | -0.32 | 1.73 | 2.03 | 0.73 | 5.65 | 0.44 | 68 |
| Dogs (yes) | -0.57 | -1.50 | 0.36 | 0.56 | 0.22 | 1.43 | 0.38 | 66 |
| Proportion worm nights* | -0.49 | -1.40 | 0.41 | 0.61 | 0.25 | 1.51 | 0.32 | 51 |
| Cattle cereals/concentrates | -0.48 | -1.50 | 0.53 | 0.62 | 0.22 | 1.70 | 0.28 | 48 |
| Palatable crops (yes) | 0.11 | -1.04 | 1.26 | 1.12 | 0.35 | 3.54 | 0.17 | 35 |
| Dairy (yes) | -0.18 | -1.20 | 0.85 | 0.84 | 0.30 | 2.33 | 0.19 | 39 |
| Palatable feed accessible (never) | 0.23 | -0.78 | 1.24 | 1.26 | 0.46 | 3.46 | 0.02 | 6 |
| Palatable feed accessible (sometimes) | 0.22 | -1.45 | 1.88 | 1.24 | 0.24 | 6.57 | 0.02 | 6 |
|  |  |  |  |  |  |  |  |  |

*continuous variables which have been standardised to mean=0, sd=0.5

**Table B – Comparison between coefficients generated using a top model set of ≤6 AICc and ≤2 AICc. From models investigating badger presence/absence and badger visitation rate. Only variables with 95% confidence intervals that did not span zero are displayed. Number of models in top model set of badger presence/absence is 93 for ≤6 AICc and 6 for ≤2 AICc. Number of models in top model set of badger visitation rate is 328 for ≤6 AICc and 20 for ≤2 AICc.**

|  |  | models AIC ≤6 | | |  | models AIC ≤2 | | |
| --- | --- | --- | --- | --- | --- | --- | --- | --- |
| response | Variable | Coefficient | coef L95% | coef  U95% |  | Coefficient | coef L95% | coef  U95% |
|  |  |  |  |  |  |  |  |  |
| **Presence / absence** | (Intercept) | -0.10 | -1.28 | 1.08 |  | -0.08 | -1.09 | 0.93 |
|  | Distance to nearest active badger sett* | -1.97 | -3.02 | -0.93 |  | -1.94 | -2.97 | -0.93 |
|  | Cattle sheds (5 or more) | 1.76 | 0.29 | 3.23 |  | 1.72 | 0.28 | 3.18 |
|  | Cattle sheds (3 to 4) | 0.16 | -1.00 | 1.32 |  | 0.18 | -0.97 | 1.33 |
|  | Feed stores* | 1.11 | 0.08 | 2.13 |  | 1.08 | 0.08 | 2.09 |
|  | House (yes) | -1.30 | -2.43 | -0.17 |  | -1.38 | -2.51 | -0.26 |
|  | Max cattle capacity* | -1.90 | -3.32 | -0.48 |  | -2.02 | -3.40 | -0.64 |
|  | Badger sett density* | 1.09 | 0.06 | 2.13 |  | 1.09 | 0.08 | 2.10 |
|  |  |  |  |  |  |  |  |  |
|  |  |  |  |  |  |  |  |  |
| **Visitation rate** | (Intercept) | -1.27 | -2.44 | -0.11 |  | -1.23 | -2.22 | -0.24 |
|  | Distance to nearest active badger sett* | -1.28 | -2.32 | -0.23 |  | -1.29 | -2.27 | -0.32 |
|  | Max cattle capacity* | 1.45 | 0.05 | 2.85 |  | 1.37 | 0.06 | 2.68 |
|  |  |  |  |  |  |  |  |  |

*continuous variables which have been standardised to mean = 0, sd = 0.5
