# Supplementary material for: Predicting badger visits to farm yards and making predictions available to farmers
Source: PLoS One. 2019 May 24;14(5):e0216953. doi: 10.1371/journal.pone.0216953 (PMC6534311; doi:10.1371/journal.pone.0216953)
Supplement: S2 Appendix — (DOCX) [file pone.0216953.s002.docx]

**S2 Appendix – Code for generating interactive app**

#load required packages

library(shiny)

library(ggplot2)

library(shinydashboard)

###########################################################################

#set the probability cutoff for presence/absence

cutoff<-0.265

#Predicted values from fat farms (training data) - values are probabilities, but expressed as percentages (ie 0.5 = 50%)

fat.pred.oc<-c(80.6428211, 57.8672580, 18.5542397, 25.6591975, 8.6391949, 15.3541319, 86.6561230, 72.5561549, 5.2565888, 21.6825720, 70.4564309, 30.1652774, 69.8184795, 16.5520561,

25.8214989, 15.2401914, 7.8365046, 51.1003876, 10.0284081, 7.9367950,16.0124396, 72.3396444, 20.6637133, 7.7226720, 8.3146376, 84.3583463, 27.4804226, 53.2556784,

72.8052544, 17.5041404, 33.7809569, 55.5362525, 55.7496025, 79.3772598, 17.1550473, 3.1284341, 39.7432833, 64.7741516, 48.5366003, 24.1242319, 64.2887729, 27.5986040,

49.8370811, 47.9363558, 7.0434431, 38.7489661, 10.4033541, 9.8510829, 51.9205376, 79.4240772, 0.4500820, 53.7796903, 36.3929107, 17.8094167, 67.7693539, 49.3563413,

90.0888756, 79.8249295, 76.5859706, 61.8764877, 9.4884754, 33.4111762, 91.2281620, 26.1890615, 55.9963805, 85.7167391, 6.9170805, 37.1322218, 10.3875396, 72.4916469,

65.6614526, 91.3449070, 16.6658894, 27.1889969, 3.5482184, 9.1610668, 40.4976246, 83.5219256, 58.6632872, 16.7986195, 5.8193174, 2.2450156, 19.9801739, 2.3235221,

48.9667589, 29.4404929, 14.7284631, 6.0625269, 9.9636540, 3.3991729, 71.0251225, 7.7279770, 20.4764755, 23.2144492, 17.7175120, 36.7762216, 36.7116610, 9.3730631,

16.8769871, 80.4670279, 0.1747020, 1.4863606, 16.5058248, 88.7184371, 46.5817785, 23.7399156, 3.6639010, 9.1737518, 3.8751725, 3.4760211, 20.2624777, 39.3306839,

10.8116806, 2.8001114, 7.0066617, 54.1559916, 3.7493725, 36.7613765, 6.3928493, 8.5654419, 3.4574934, 23.3017730, 41.9820063, 2.5248655, 1.6555085, 5.8885703,

17.0568562, 5.7248180, 4.4089390, 8.7923588, 91.5004966, 89.7630224, 26.3638342, 44.5727704, 21.8943157, 5.9667468, 6.6150682, 59.9568804, 65.8166560, 17.0126028,

87.6912306, 12.0627785, 2.1184876, 0.2326468, 32.5101017, 78.1199767, 18.3227301, 3.4198856, 46.0746735, 6.4559469, 30.6093010, 4.1496762, 5.8636821, 7.1989754,

21.9544160)

#Predicted values from 'husbandry' farms (test data) - values are probabilities, but expressed as percentages

husb.pred.oc<-c(31.286494, 45.333683, 3.270127, 14.305171, 91.667740, 37.835768, 47.908587, 70.407086, 10.067778, 1.956828, 15.750780, 59.337658, 94.613250, 29.079064, 47.375487,

68.526278, 46.391892, 8.842404, 2.165202, 6.898998, 2.513814, 89.772951, 5.258705, 2.072403, 11.450011, 31.358149, 7.363625, 1.455998, 15.868026, 3.736164,

30.169463, 3.182660, 12.012448, 1.265409, 1.997812, 4.154979, 3.195203, 3.081892, 58.305803, 7.761090)

#Combine data sets

husb.pred.oc<-husb.pred/(husb.pred+1)*100

farm.pred<-c(fat.pred.oc,husb.pred.oc)

#the app displays risk scores relative to other farms. This calculates what % of the farms in the data set above are below the cutoff value chosen

percutoff<-length(farm.pred[farm.pred<=(cutoff*100)])/length((farm.pred))*100 #38.46 so a predicted value >38.46% = high risk.

#Sort out data for high/low plot

yvar<-rep(seq(1,10),each=10)

xvar<-rep(seq(1,10),times=10)

plot.dat.low<-data.frame(cbind(yvar,xvar))

plot.dat.low$cat<-c(rep("a",times=29),rep("b",times=71))

plot.dat.high<-data.frame(cbind(yvar,xvar))

plot.dat.high$cat<-c(rep("a",times=75),rep("b",times=25))

###########################################################################

server <- function(input, output) {#Creates a reactive value which is the predicted value given the user inputs

pred.value<- reactive({#first input variables are converted and standardised so that they can be multiplied by the model coefficients

#first input variables are converted and standardised so that they can be multiplied by the model coefficients

#first input variables are converted and standardised so that they can be multiplied by the model coefficients

sheds.3to4<-ifelse(input$cattlesheds<5 & input$cattlesheds>2,1,0) #categorical - 3 to 4 cattle sheds

sheds.5ormore<-ifelse(input$cattlesheds>4,1,0) # categorical 5 or more cattle sheds

house.no<-ifelse(input$houseyn=="1",1,0) # categorical house yes or no

#Continous varialbles were standardised for the GLM - input varaibles therefore need to be standardised accordingly

#...this is doen by subtracting the mean and dividing by 2Xsd

st.f.stores<-(input$feedstores-1.729032)/(2*1.300897)

st.max.cattle<-(input$cattle-178.0774)/(2*154.9194)

st.no.sett<-((input$setts/0.7853982)-6.132458)/(2*6.232756)

#the input for sett distqance is categorical - this is converted to continous value of 1-6...

sett.dist.no<-ifelse(input$distance=="1",1,

ifelse(input$distance=="2",2,ifelse(input$distance=="3",3,

ifelse(input$distance=="4",4,ifelse(input$distance=="5",5,6)))))

#...and the standardised

st.sett.dist<-(sett.dist.no-3.703226)/(2* 1.636333)

#predicted probability of badger visits calcuated using input varaibles and model coefficients

pred<-exp(-0.09974769+

(sheds.5ormore*1.74082841)+

(sheds.3to4*0.14682129)+

(st.f.stores*1.09458284)+

(house.no*-1.29451750)+

(st.max.cattle*-1.88384875)+

(st.no.sett*1.07816714)+

(st.sett.dist*-1.98708242)

)

(pred/(pred+1))*100 # then converted to a percentage

})

##############################################################################################################################

#The following code generates the high risk/low risk bar with the arrow

output$plot.test<-renderPlot({

mydf <- data.frame(id = rep(1, 101), sales = 0:100)

score<-length(farm.pred[farm.pred<=pred.value()])/length((farm.pred))*100

ggplot(mydf) +

geom_tile(aes(y = 1, x=sales, fill = sales)) +

geom_rect(ymin=0.5,ymax=1.5,xmin=0.5,xmax=100.5,size=1,alpha=0,color="black")+ #outline of the scale

scale_y_continuous(limits=c(0,2),breaks=1)+ #controls the height of the plot

scale_x_continuous(limits=c(0,110))+

scale_fill_gradient2(low = 'blue', mid = 'white', high = 'red', midpoint = percutoff) + #controls colour split of plot

geom_segment(aes(y = 0.5, x = percutoff, yend = 1.5, xend = percutoff),size=2)+

geom_segment(aes(y = 2, x = score, yend = 1.52, xend = score),color="red4",size=3,arrow = arrow(length = unit(0.5, "cm")),lineend = "square")+ #creates the arrow

annotate("text",y=1.85,x=score+10,label=paste(round(score),"%"),size=10)+

annotate("text", y=1, x=percutoff/2, label= "Low Risk of badger visits",size=10)+

annotate("text", y=1, x=((100-percutoff)/2)+percutoff, label= "High Risk of badger visits",size=10)+

theme(legend.position="none",

axis.title.x=element_blank(),

axis.text.x=element_blank(),

axis.ticks.y=element_blank(),

axis.title.y=element_blank(),

axis.text.y=element_blank(),

axis.ticks.x=element_blank(),

axis.line = element_blank(),

panel.grid.major = element_blank(),

panel.grid.minor = element_blank(),

panel.background = element_blank())

})

#Several interactive text objects are also created to display in the app

output$text1 <-renderText({round(pred.value())})#rounded predicted value

risk<-reactive({round

(length(farm.pred[farm.pred<=pred.value()])/length((farm.pred))*100)})#calculatesd the % of farms (from our data) with lower risk scores

output$text2<-renderText({

risk.cat<-ifelse(risk()>percutoff,"High","Low")

paste(risk.cat," risk of badger visits - what does this mean?")

})#Displays high or low risk, depending on score generated and cutoff chosen

output$text3<-renderText({

risk.cat<-ifelse(risk()>percutoff,"High","Low")

paste("Based on the characteristics above, your farm has a risk score of ", round(risk()), "%. This means that out of 100 farms in our study you have a risk score which higher than ", round(risk()), "of them. This places you in the ", risk.cat, "risk of badger visits category.")

})

output$text4<-renderText({

ifelse(risk()>percutoff,"Of the farms analysed in our study 75% (3 in 4) of high risk farms had badgers visiting their yards and buildings and 25% (1 in 4) experienced no visits. Farms in the high risk category which experienced visits, also experienced them more frequently (on more nights) than those in the low risk category","Of the farms analysed in our study 29% (just under 1 in 3) of low risk farms had badgers visiting their yards and buildings and 71% (just over 2 in 3) experienced no visits. Farms in the low risk category which experienced visits, also experienced them less frequently (on fewer nights) than those in the high risk category ")

})#interactive text depending on whether the farm is classe as high or low

output$riskcat.text<-renderText({

risk.cat<-ifelse(risk()>percutoff,"High","Low")

})

# this code generates the figure at the bottom of the app.

output$risk.plot<-renderPlot({

if(risk()>percutoff){plot.dat<-plot.dat.high}

if(risk()<=percutoff){plot.dat<-plot.dat.low}

plot.title<-ifelse(risk()>percutoff,"How many 'high risk' farms experience visits?","How many 'low risk' farms experience visits?")

p.text1.y<-ifelse(risk()>percutoff,4.5,2)

p.text2.y<-ifelse(risk()>percutoff,9.5,7.5)

ggplot(plot.dat, aes(x=xvar, y=yvar, color=cat))+

geom_point(shape=16, size=10)+

annotate("text",y=p.text1.y,x=5,label="Badger visits",size=8)+

annotate("text",y=p.text2.y,x=5,label="No badger visits",size=8)+

theme(legend.position="none",

plot.title=element_text(size=20),

axis.title.x=element_blank(),

axis.text.x=element_blank(),

axis.ticks.y=element_blank(),

axis.title.y=element_blank(),

axis.text.y=element_blank(),

axis.ticks.x=element_blank(),

axis.line = element_blank(),

panel.grid.major = element_blank(),

panel.grid.minor = element_blank(),

panel.border = element_blank(),

panel.background = element_blank())

})

}

dash_sidebar<-dashboardSidebar(disable=TRUE)

dash_body<-dashboardBody(

fluidRow(column(12,offset=0,h1(" Badger Activity Farm Assessment Tool"))),

fluidRow(column(12,offset=0,

h2("About this tool"),p("The European badger (Meles meles) acts a potential source of TB infection in cattle. Badgers may enter farm yards and buildings, bringing them into close contact cattle. Badgers may also contaminate cattle feed, water or the farm environment, which is a potential disease risk. This interactive tool aims to identify farms at risk of badger visits. The risk score is based on a statistical analysis of 155 farms in the South-West, 40% of which experienced badger visits (recorded on cameras). Enter the characteristics of your farm using the buttons and the risk score for your farm (and what this means) will be displayed below."

),img(height=203,width=1152, src="badgers.png"))),

fluidRow(column(12, offset=0,h2("Step 1 - Enter your farm characterstics"))),

fluidRow(column(6,offset=0,

h3("Q1 - Cattle sheds"),

p("A cattle shed is any building where cattle are regularly housed. In our study area most farms had 3 cattle sheds (range 0-14)"),

sliderInput("cattlesheds","How many cattle sheds do you have?",min=0,max=20,value=1),

h3("Q2 - Size of your cattle housing"),

p("Badger visitation is related to the number of cattle that can be housed at the farm. In our study area most farms could house a maximum of 140 cattle (range 12-1100)"),

sliderInput("cattle","What is the maximum number of cattle you house in your buildings?",min=0,max=2000,value=100, step=10),

h3("Q3 - Feedstores"),

p("A feedstore is a any building where feed is regularly stored and this includes silage or feed clamps. In our study area most farms had only one feed store (range 0-6)"),

sliderInput("feedstores","How many feed stores do you have?",min=0,max=10,value=1)),

column(6,offset=0,

h3("Q4 - Presence of a house or dwelling"),

p("These includes any buildings where people are living/sleeping regularly on site (ie in or next to the farmyard)."),

radioButtons("houseyn", "Is there a house/dwelling in your farm?",choices = list("Yes" = 1, "No" = 2),selected = 1),

h3("Q5 - Badger setts"),

p("Badger setts are burrow systems and can have anywhere between 1 and 40+ entrance holes. For advice on badger field signs visit the ",a(href="http://www.tbhub.co.uk/badger-biosecurity-factsheets/", "TBhub website.")),

sliderInput("setts","How many badger setts are located within 500m of your farm yard?",min=0,max=30,value=1),

h3("Q6 - Distance to nearest active sett"),

p("Active badger setts have entrances which are generally clear of debris (leaves or sticks) and have fresh spoil, prints, or other field signs. For advice on badger field signs visit the ",a(href="http://www.tbhub.co.uk/badger-biosecurity-factsheets/", "TBhub website.")),

radioButtons("distance","How close (to nearest 100m) is the nearest active badger sett to your farm?",

choices = list("0-100m" = 1, "100-200m" = 2,"200-300m"=3, "300-400m"=4, "400-500m"=5,"more than 500m"=6),selected = 3))),

fluidRow(box(width=12,h2("Step 2 - The risk score for your farm"),

h3(textOutput("text3")), #state the % and risk score for the farm

plotOutput("plot.test", height = "300px",width="1000px"), #risk plot

h2(textOutput("text2")),

h3(textOutput("text4")),

plotOutput("risk.plot", width="300px", height="300px")

)),

fluidRow(column(12,h2("Step 3 - What next? How to reduce badger activity in farm yards"),

h3("If you are in an area with high levels of TB, or of you think there is a local wildlife risk then it advisable that measures are taken to reduce badger activity in your yard and buildings. Research has shown that relative simple measures including sheet fencing, modified gates, doors and feed stores can significantly reduce badger activity in farm yards. For practical advice on measures to reduce badger activity visit ",

a(href="http://www.tbhub.co.uk/biosecurity/biosecurity-factsheets/", "www.TBhub.co.uk"), "Trail cameras or 'camera traps' can be used to confirm badger activity in farm yards and to identify specific areas of the farm where biosecurity measures can be applied.", "For more information on using camera traps to assess badger activity click ",

a(href="http://www.tbhub.co.uk/badger-biosecurity-factsheets/", "here"))

)

),

fluidRow(img(height=203,width=1152, src="measures.png"))

)

#launch the app

shinyApp(ui = dashboardPage(skin="blue",

dashboardHeader(disable = TRUE),

dash_sidebar,dash_body),

server = server)

########################################################
