# Supplementary material for: Predicting badger visits to farm yards and making predictions available to farmers
Source: PLoS One. 2019 May 24;14(5):e0216953. doi: 10.1371/journal.pone.0216953 (PMC6534311; doi:10.1371/journal.pone.0216953)
Supplement: S3 Appendix — (DOCX) [file pone.0216953.s003.docx]

**S3 Appendix – data for analyses**

| **Variable** |  | **description** |
| --- | --- | --- |
| data | A | Training (155 farms monitored in this study) or test (40 setts from Judge et al. 2011) |
| farm.number | B | Id number of farm |
| start.date.D | C | Start date of monitoring |
| year | D | Year of survey |
| central.month | E | Central month of survey |
| season | F | season (spring / summer / autum) |
| feed.stores | G | Number of feed stroes on the farm |
| max.cattle.capacity | H | Max number cattle housed on farm |
| lights | I | Presence of lights on a night |
| house.dwelling | J | presence of house/dwelling on farm |
| edible.crops | K | presence of edible/crops on farm |
| cattle_cereal_or_concnetrates | L | Presence of cattle_cereal_or_concnetrates |
| edible_feed_accesible | M | Edible number feed housed on farm y/n |
| cattle.sheds.cat | N | Number of cattle sheds |
| sett.density | O | Density of badger setts |
| TB.restrictions | P | Under TB restrictions y/n during survey period |
| number.setts | Q | Number of setts |
| area.surveyed | R | Area surveyed for badger field signs |
| nearest.active.sett.dist | S | Distance to nearest active badger sett |
| no.nights.badger.visits | T | Number nights badgers recorded on camera |
| any.badger.visits | U | Badger visits on camera y/n |
| n.nights.surveyed | V | Number of nights surveyed |
| prop.worm.nights | W | Proportion of survey nights classed as worm nights |
| dairy | X | Presence of dairy on farm |
| dog | Y | Presence of dogs on farm |

*Only significant variables used for prediction are included for the 40 test setts

7

| A | B | C | D | E | F | G | H | I | J | K | L | M | N | O | P | Q | R | S | T | U | V | W | X | Y |
| --- | --- | --- | --- | --- | --- | --- | --- | --- | --- | --- | --- | --- | --- | --- | --- | --- | --- | --- | --- | --- | --- | --- | --- | --- |
| training | 57 | 27/07/2012 | 2012 | August | late.summer.autumn | 1 | 90 | 0 | 0 | no | no | never | sheds.upto.2 | 18.56763926 | 1 | 14 | 0.754 | 199.1557563 | 1 | 1 | 32 | 71.9 | no | N |
| training | 60 | 31/07/2012 | 2012 | August | late.summer.autumn | 2 | 12 | 0 | 1 | yes | no | never | sheds.upto.2 | 3.94218134 | 0 | 3 | 0.761 | 153.9158525 | 0 | 0 | 30 | 83.3 | no | Y |
| training | 66 | 07/08/2012 | 2012 | August | late.summer.autumn | 0 | 25 | 0 | 0 | no | no | never | sheds.upto.2 | 14.01273885 | 0 | 11 | 0.785 | 110.6072367 | 7 | 1 | 29 | 69 | no | N |
| training | 85 | 03/05/2013 | 2013 | May | spring | 1 | 20 | 0 | 1 | yes | yes | allyear | sheds.upto.2 | 3.205128205 | 0 | 2 | 0.624 | 121.03 | 0 | 0 | 39 | 46.2 | no | N |
| training | 111 | 13/06/2013 | 2013 | June | summer | 2 | 160 | 1 | 1 | yes | yes | never | sheds.upto.2 | 6.884681583 | 1 | 4 | 0.581 | 318.85 | 0 | 0 | 29 | 58.6 | yes | N |
| training | 120 | 04/07/2013 | 2013 | July | summer | 1 | 80 | 0 | 1 | yes | yes | allyear | sheds.upto.2 | 1.597444089 | 0 | 1 | 0.626 | 424.34 | 0 | 0 | 33 | 33.3 | yes | N |
| training | 141 | 14/08/2013 | 2013 | August | late.summer.autumn | 0 | 80 | 0 | 0 | yes | yes | never | sheds.upto.2 | 12.7388535 | 1 | 6 | 0.471 | 30.44 | 2 | 1 | 35 | 57.1 | no | N |
| training | 7 | 13/04/2012 | 2012 | April | spring | 1 | 40 | 0 | 0 | yes | yes | sometimes | sheds.upto.2 | 4.010695187 | 0 | 3 | 0.748 | 21.19992279 | 4 | 1 | 28 | 82.1 | no | N |
| training | 11 | 17/04/2012 | 2012 | May | spring | 1 | 50 | 0 | 1 | no | no | never | sheds.upto.2 | 15.9453303 | 0 | 7 | 0.439 | 119.003411 | 22 | 1 | 29 | 89.7 | no | Y |
| training | 13 | 20/04/2012 | 2012 | May | spring | 1 | 35 | 0 | 1 | no | no | never | sheds.upto.2 | 14.53957997 | 0 | 9 | 0.619 | 100.988129 | 0 | 0 | 31 | 83.9 | no | Y |
| training | 24 | 03/05/2012 | 2012 | May | spring | 1 | 40 | 1 | 1 | no | yes | never | sheds.upto.2 | 4.608294931 | 0 | 3 | 0.651 | 501 | 0 | 0 | 34 | 44.1 | no | N |
| training | 33 | 14/05/2012 | 2012 | May | spring | 0 | 20 | 0 | 0 | yes | no | never | sheds.upto.2 | 2.547770701 | 0 | 2 | 0.785 | 246.049545 | 11 | 1 | 32 | 50 | no | N |
| training | 41 | 28/05/2012 | 2012 | June | summer | 2 | 50 | 0 | 1 | no | no | never | sheds.upto.2 | 7.8125 | 0 | 6 | 0.768 | 117.8772188 | 3 | 1 | 32 | 75 | no | Y |
| training | 43 | 29/05/2012 | 2012 | June | summer | 0 | 25 | 0 | 1 | no | no | never | sheds.upto.2 | 15.93625498 | 0 | 4 | 0.251 | 238.4431357 | 0 | 0 | 35 | 85.7 | no | Y |
| training | 56 | 27/07/2012 | 2012 | August | late.summer.autumn | 0 | 90 | 0 | 1 | no | no | never | sheds.upto.2 | 20.26049204 | 1 | 14 | 0.691 | 228.910253 | 0 | 0 | 32 | 71.9 | no | Y |
| training | 58 | 27/07/2012 | 2012 | August | late.summer.autumn | 2 | 50 | 0 | 1 | no | yes | never | sheds.upto.2 | 17.01570681 | 2 | 13 | 0.764 | 101.363199 | 1 | 1 | 32 | 71.9 | no | N |
| training | 72 | 17/08/2012 | 2012 | September | late.summer.autumn | 1 | 20 | 0 | 0 | no | no | never | sheds.upto.2 | 15.41425819 | 0 | 8 | 0.519 | 122.1072332 | 4 | 1 | 34 | 41.2 | no | N |
| training | 74 | 23/08/2012 | 2012 | September | late.summer.autumn | 2 | 40 | 0 | 1 | yes | no | never | sheds.upto.2 | 2.985074627 | 0 | 2 | 0.67 | 313.2930094 | 0 | 0 | 32 | 40.6 | no | N |
| training | 78 | 18/04/2013 | 2013 | May | spring | 0 | 35 | 0 | 0 | yes | yes | sometimes | sheds.upto.2 | 12.7388535 | 0 | 10 | 0.785 | 139.25 | 0 | 0 | 29 | 41.4 | no | N |
| training | 91 | 09/05/2013 | 2013 | May | spring | 1 | 200 | 0 | 0 | yes | yes | never | sheds.upto.2 | 3.821656051 | 1 | 3 | 0.785 | 52.94 | 0 | 0 | 29 | 44.8 | no | N |
| training | 92 | 09/05/2013 | 2013 | May | spring | 1 | 200 | 0 | 0 | yes | yes | never | sheds.upto.2 | 0 | 1 | 0 | 0.641 | 501 | 2 | 1 | 29 | 44.8 | no | N |
| training | 93 | 09/05/2013 | 2013 | May | spring | 1 | 70 | 0 | 1 | no | no | allyear | sheds.upto.2 | 5.586592179 | 0 | 4 | 0.716 | 332.09 | 0 | 0 | 29 | 44.8 | no | Y |
| training | 100 | 23/05/2013 | 2013 | June | summer | 1 | 30 | 0 | 1 | no | yes | allyear | sheds.upto.2 | 20.93397746 | 0 | 13 | 0.621 | 112.65 | 1 | 1 | 32 | 53.1 | no | N |
| training | 101 | 24/05/2013 | 2013 | June | summer | 1 | 620 | 1 | 1 | yes | yes | allyear | sheds.upto.2 | 0 | 0 | 0 | 0.785 | 501 | 0 | 0 | 31 | 61.3 | no | Y |
| training | 104 | 30/05/2013 | 2013 | June | summer | 1 | 30 | 0 | 0 | no | yes | never | sheds.upto.2 | 5.547850208 | 0 | 4 | 0.721 | 96.29 | 19 | 1 | 31 | 54.8 | yes | N |
| training | 105 | 31/05/2013 | 2013 | June | summer | 0 | 16 | 0 | 0 | yes | no | never | sheds.upto.2 | 5.095541401 | 0 | 4 | 0.785 | 384.17 | 0 | 0 | 31 | 58.1 | no | N |
| training | 114 | 27/06/2013 | 2013 | July | summer | 0 | 83 | 0 | 1 | yes | yes | allyear | sheds.upto.2 | 0 | 0 | 0 | 0.785 | 501 | 0 | 0 | 29 | 20.7 | yes | Y |
| training | 121 | 04/07/2013 | 2013 | July | summer | 1 | 160 | 0 | 1 | yes | yes | allyear | sheds.upto.2 | 3.086419753 | 0 | 1 | 0.324 | 501 | 0 | 0 | 35 | 37.1 | no | N |
| training | 122 | 05/07/2013 | 2013 | July | summer | 0 | 80 | 0 | 0 | yes | yes | never | sheds.upto.2 | 4.379562044 | 1 | 3 | 0.685 | 402.68 | 0 | 0 | 32 | 37.5 | yes | N |
| training | 123 | 05/07/2013 | 2013 | July | summer | 1 | 80 | 1 | 0 | yes | no | never | sheds.upto.2 | 2.547770701 | 0 | 2 | 0.785 | 383.75 | 1 | 1 | 32 | 43.8 | no | N |
| training | 132 | 17/07/2013 | 2013 | August | late.summer.autumn | 0 | 33 | 0 | 0 | no | yes | never | sheds.upto.2 | 11.87648456 | 0 | 5 | 0.421 | 63.43 | 2 | 1 | 30 | 50 | no | N |
| training | 134 | 26/07/2013 | 2013 | August | late.summer.autumn | 1 | 70 | 0 | 0 | yes | yes | sometimes | sheds.upto.2 | 3.081664099 | 0 | 2 | 0.649 | 346.88 | 0 | 0 | 28 | 50 | no | N |
| training | 138 | 31/07/2013 | 2013 | August | late.summer.autumn | 1 | 30 | 0 | 0 | yes | no | never | sheds.upto.2 | 7.547169811 | 0 | 4 | 0.53 | 315.72 | 4 | 1 | 30 | 50 | no | Y |
| training | 149 | 21/08/2013 | 2013 | September | late.summer.autumn | 0 | 60 | 0 | 0 | yes | yes | never | sheds.upto.2 | 7.936507937 | 0 | 5 | 0.63 | 350.37 | 0 | 0 | 35 | 40 | no | N |
| training | 2 | 12/04/2012 | 2012 | April | spring | 2 | 110 | 0 | 1 | yes | no | sometimes | sheds.upto.2 | 1.721170396 | 0 | 1 | 0.581 | 91.31834623 | 0 | 0 | 29 | 82.8 | no | Y |
| training | 22 | 02/05/2012 | 2012 | May | spring | 2 | 50 | 0 | 1 | yes | no | never | sheds.upto.2 | 19.30501931 | 1 | 15 | 0.777 | 229.8525701 | 3 | 1 | 29 | 41.4 | no | N |
| training | 25 | 03/05/2012 | 2012 | May | spring | 1 | 100 | 1 | 1 | yes | no | never | sheds.upto.2 | 2.617801047 | 0 | 2 | 0.764 | 477.0280933 | 0 | 0 | 34 | 44.1 | no | N |
| training | 34 | 16/05/2012 | 2012 | May | spring | 1 | 65 | 0 | 0 | yes | yes | never | sheds.upto.2 | 6.775067751 | 0 | 5 | 0.738 | 197.24 | 0 | 0 | 30 | 50 | no | Y |
| training | 38 | 22/05/2012 | 2012 | June | summer | 3 | 100 | 0 | 0 | yes | no | never | sheds.upto.2 | 5.154639175 | 0 | 4 | 0.776 | 356.4510248 | 2 | 1 | 31 | 61.3 | no | Y |
| training | 39 | 24/05/2012 | 2012 | June | summer | 3 | 140 | 1 | 1 | yes | yes | allyear | sheds.upto.2 | 1.709401709 | 1 | 1 | 0.585 | 178.7981972 | 14 | 1 | 32 | 68.8 | yes | N |
| training | 42 | 29/05/2012 | 2012 | June | summer | 1 | 50 | 1 | 1 | yes | yes | allyear | sheds.upto.2 | 8.771929825 | 0 | 3 | 0.342 | 348.4155353 | 0 | 0 | 31 | 77.4 | no | Y |
| training | 45 | 12/06/2012 | 2012 | June | summer | 2 | 60 | 0 | 1 | yes | yes | never | sheds.upto.2 | 0 | 0 | 0 | 0.785 | 501 | 0 | 0 | 29 | 93.1 | no | N |
| training | 50 | 28/06/2012 | 2012 | July | summer | 1 | 100 | 0 | 1 | no | no | never | sheds.upto.2 | 17.94453507 | 0 | 11 | 0.613 | 97.65534716 | 5 | 1 | 27 | 85.2 | no | N |
| training | 52 | 25/07/2012 | 2012 | August | late.summer.autumn | 2 | 75 | 0 | 0 | yes | no | never | sheds.upto.2 | 2.90275762 | 0 | 2 | 0.689 | 354.0257192 | 4 | 1 | 29 | 69 | no | N |
| training | 53 | 25/07/2012 | 2012 | August | late.summer.autumn | 1 | 60 | 0 | 1 | no | yes | never | sheds.upto.2 | 14.21800948 | 0 | 6 | 0.422 | 348.8222525 | 0 | 0 | 29 | 62.1 | no | Y |
| training | 54 | 26/07/2012 | 2012 | August | late.summer.autumn | 1 | 200 | 0 | 1 | yes | yes | never | sheds.upto.2 | 5.540166205 | 1 | 4 | 0.722 | 273.7114024 | 0 | 0 | 29 | 75.9 | no | Y |
| training | 55 | 26/07/2012 | 2012 | August | late.summer.autumn | 2 | 200 | 0 | 0 | yes | yes | never | sheds.upto.2 | 4.310344828 | 1 | 3 | 0.696 | 198.3262064 | 8 | 1 | 29 | 75.9 | no | N |
| training | 65 | 07/08/2012 | 2012 | August | late.summer.autumn | 0 | 120 | 0 | 1 | yes | yes | sometimes | sheds.upto.2 | 25.47770701 | 0 | 20 | 0.785 | 267.9635537 | 1 | 1 | 29 | 69 | no | Y |
| training | 71 | 15/08/2012 | 2012 | August | late.summer.autumn | 3 | 50 | 0 | 1 | yes | no | never | sheds.upto.2 | 10.79913607 | 0 | 5 | 0.463 | 256.8251743 | 13 | 1 | 30 | 53.3 | no | N |
| training | 73 | 20/08/2012 | 2012 | September | late.summer.autumn | 3 | 230 | 1 | 1 | yes | yes | never | sheds.upto.2 | 4.137931034 | 1 | 3 | 0.725 | 328.5208592 | 0 | 0 | 31 | 32.3 | no | N |
| training | 77 | 17/04/2013 | 2013 | May | spring | 2 | 40 | 0 | 0 | yes | no | never | sheds.upto.2 | 1.314060447 | 0 | 1 | 0.761 | 481.12 | 5 | 1 | 29 | 31 | yes | N |
| training | 79 | 19/04/2013 | 2013 | May | spring | 1 | 130 | 0 | 0 | no | no | never | sheds.upto.2 | 6.791171477 | 0 | 4 | 0.589 | 249.7 | 1 | 1 | 31 | 41.9 | yes | N |
| training | 80 | 22/04/2013 | 2013 | May | spring | 1 | 140 | 1 | 1 | yes | yes | never | sheds.upto.2 | 7.643312102 | 0 | 6 | 0.785 | 333.46 | 32 | 1 | 32 | 43.8 | no | N |
| training | 86 | 07/05/2013 | 2013 | May | spring | 1 | 150 | 0 | 1 | yes | yes | sometimes | sheds.upto.2 | 2.570694087 | 0 | 1 | 0.389 | 190.47 | 10 | 1 | 30 | 60 | no | N |
| training | 102 | 30/05/2013 | 2013 | June | summer | 1 | 300 | 1 | 1 | no | yes | allyear | sheds.upto.2 | 2.781641168 | 0 | 2 | 0.719 | 501 | 0 | 0 | 31 | 54.8 | yes | N |
| training | 106 | 03/06/2013 | 2013 | June | summer | 2 | 50 | 0 | 0 | yes | yes | never | sheds.upto.2 | 0 | 0 | 0 | 0.466 | 501 | 0 | 0 | 35 | 34.3 | no | N |
| training | 107 | 03/06/2013 | 2013 | June | summer | 2 | 200 | 1 | 1 | yes | yes | never | sheds.upto.2 | 1.742160279 | 0 | 1 | 0.574 | 501 | 0 | 0 | 35 | 40 | yes | Y |
| training | 108 | 04/06/2013 | 2013 | June | summer | 2 | 12 | 0 | 1 | yes | yes | sometimes | sheds.upto.2 | 0 | 1 | 0 | 0.568 | 501 | 0 | 0 | 31 | 58.1 | no | N |
| training | 113 | 14/06/2013 | 2013 | June | summer | 1 | 60 | 0 | 1 | yes | no | sometimes | sheds.upto.2 | 3.210272873 | 0 | 2 | 0.623 | 400.03 | 0 | 0 | 31 | 35.5 | no | Y |
| training | 116 | 27/06/2013 | 2013 | July | summer | 1 | 100 | 0 | 0 | yes | yes | allyear | sheds.upto.2 | 2.547770701 | 0 | 2 | 0.785 | 261.14 | 12 | 1 | 29 | 20.7 | no | N |
| training | 117 | 27/06/2013 | 2013 | July | summer | 1 | 450 | 1 | 1 | yes | yes | never | sheds.upto.2 | 2.980625931 | 1 | 2 | 0.671 | 264.76 | 0 | 0 | 29 | 20.7 | yes | N |
| training | 124 | 08/07/2013 | 2013 | July | summer | 1 | 170 | 1 | 1 | yes | yes | never | sheds.upto.2 | 0 | 0 | 0 | 0.525 | 501 | 0 | 0 | 35 | 48.6 | no | Y |
| training | 130 | 11/07/2013 | 2013 | July | summer | 2 | 50 | 0 | 1 | yes | no | never | sheds.upto.2 | 2.114164905 | 1 | 1 | 0.473 | 501 | 0 | 0 | 29 | 48.3 | yes | N |
| training | 139 | 07/08/2013 | 2013 | August | late.summer.autumn | 1 | 26 | 0 | 1 | yes | no | never | sheds.upto.2 | 4.636785162 | 0 | 3 | 0.647 | 26.96 | 21 | 1 | 34 | 32.4 | no | N |
| training | 140 | 08/08/2013 | 2013 | August | late.summer.autumn | 1 | 55 | 0 | 1 | yes | yes | sometimes | sheds.upto.2 | 1.872659176 | 0 | 1 | 0.534 | 379.15 | 0 | 0 | 29 | 27.6 | no | N |
| training | 146 | 16/08/2013 | 2013 | September | late.summer.autumn | 0 | 100 | 0 | 0 | yes | yes | allyear | sheds.upto.2 | 13.19261214 | 0 | 5 | 0.379 | 191.85 | 22 | 1 | 35 | 60 | no | N |
| training | 147 | 21/08/2013 | 2013 | September | late.summer.autumn | 1 | 260 | 0 | 1 | yes | yes | never | sheds.upto.2 | 3.016591252 | 0 | 2 | 0.663 | 185.4 | 0 | 0 | 35 | 48.6 | no | Y |
| training | 6 | 13/04/2012 | 2012 | April | spring | 4 | 80 | 1 | 1 | yes | yes | sometimes | sheds.3to4 | 0 | 0 | 0 | 0.785 | 501 | 1 | 1 | 28 | 82.1 | yes | Y |
| training | 18 | 26/04/2012 | 2012 | May | spring | 4 | 130 | 1 | 1 | yes | no | never | sheds.3to4 | 9.708737864 | 0 | 6 | 0.618 | 307.970416 | 0 | 0 | 29 | 62.1 | yes | N |
| training | 23 | 02/05/2012 | 2012 | May | spring | 1 | 140 | 1 | 1 | yes | yes | never | sheds.3to4 | 1.582278481 | 1 | 1 | 0.632 | 234.7121041 | 0 | 0 | 29 | 41.4 | yes | Y |
| training | 30 | 11/05/2012 | 2012 | May | spring | 1 | 250 | 0 | 1 | no | yes | never | sheds.3to4 | 14.01273885 | 0 | 11 | 0.785 | 305.4345061 | 0 | 0 | 31 | 38.7 | yes | N |
| training | 32 | 14/05/2012 | 2012 | May | spring | 4 | 90 | 1 | 1 | yes | yes | allyear | sheds.3to4 | 1.865671642 | 0 | 1 | 0.536 | 273.4323267 | 1 | 1 | 32 | 50 | no | Y |
| training | 35 | 17/05/2012 | 2012 | June | summer | 1 | 200 | 0 | 1 | yes | yes | never | sheds.3to4 | 3.100775194 | 1 | 2 | 0.645 | 263.49 | 0 | 0 | 32 | 46.9 | no | N |
| training | 37 | 22/05/2012 | 2012 | June | summer | 1 | 100 | 1 | 1 | yes | no | never | sheds.3to4 | 9.641873278 | 0 | 7 | 0.726 | 237.4134758 | 17 | 1 | 31 | 61.3 | no | Y |
| training | 47 | 14/06/2012 | 2012 | June | summer | 1 | 100 | 1 | 1 | yes | yes | allyear | sheds.3to4 | 3.597122302 | 0 | 1 | 0.278 | 488.12 | 0 | 0 | 33 | 90.9 | yes | N |
| training | 49 | 21/06/2012 | 2012 | July | summer | 1 | 120 | 0 | 1 | yes | no | never | sheds.3to4 | 2.547770701 | 0 | 2 | 0.785 | 51.10065817 | 13 | 1 | 34 | 82.4 | no | Y |
| training | 63 | 03/08/2012 | 2012 | August | late.summer.autumn | 3 | 150 | 1 | 0 | yes | yes | allyear | sheds.3to4 | 12.7388535 | 0 | 10 | 0.785 | 161.8974539 | 0 | 0 | 32 | 75 | yes | N |
| training | 68 | 09/08/2012 | 2012 | August | late.summer.autumn | 3 | 200 | 1 | 1 | yes | yes | allyear | sheds.3to4 | 5.657708628 | 0 | 4 | 0.707 | 282.2908455 | 0 | 0 | 29 | 82.8 | no | Y |
| training | 94 | 13/05/2013 | 2013 | May | spring | 2 | 300 | 1 | 1 | yes | yes | never | sheds.3to4 | 2.547770701 | 1 | 2 | 0.785 | 141.11 | 0 | 0 | 29 | 51.7 | yes | N |
| training | 97 | 17/05/2013 | 2013 | June | summer | 1 | 400 | 0 | 1 | no | yes | never | sheds.3to4 | 22.00825309 | 0 | 16 | 0.727 | 152.68 | 0 | 0 | 38 | 50 | no | Y |
| training | 109 | 04/06/2013 | 2013 | June | summer | 0 | 80 | 1 | 1 | yes | yes | never | sheds.3to4 | 1.808318264 | 0 | 1 | 0.553 | 501 | 0 | 0 | 31 | 58.1 | no | Y |
| training | 110 | 10/06/2013 | 2013 | June | summer | 4 | 350 | 1 | 1 | yes | yes | allyear | sheds.3to4 | 0 | 1 | 0 | 0.625 | 501 | 0 | 0 | 32 | 37.5 | yes | Y |
| training | 112 | 13/06/2013 | 2013 | June | summer | 1 | 110 | 1 | 1 | yes | yes | allyear | sheds.3to4 | 3.012048193 | 0 | 1 | 0.332 | 127.6 | 1 | 1 | 29 | 44.8 | yes | N |
| training | 119 | 04/07/2013 | 2013 | July | summer | 0 | 90 | 1 | 1 | yes | yes | never | sheds.3to4 | 1.540832049 | 0 | 1 | 0.649 | 476.12 | 0 | 0 | 33 | 33.3 | yes | N |
| training | 125 | 09/07/2013 | 2013 | July | summer | 0 | 200 | 1 | 1 | yes | no | never | sheds.3to4 | 0 | 0 | 0 | 0.519 | 501 | 0 | 0 | 31 | 45.2 | no | N |
| training | 126 | 09/07/2013 | 2013 | July | summer | 1 | 50 | 1 | 1 | no | no | never | sheds.3to4 | 0 | 0 | 0 | 0.672 | 501 | 0 | 0 | 31 | 45.2 | no | Y |
| training | 128 | 10/07/2013 | 2013 | July | summer | 1 | 55 | 0 | 1 | no | no | never | sheds.3to4 | 0 | 0 | 0 | 0.675 | 501 | 0 | 0 | 30 | 46.7 | no | N |
| training | 136 | 29/07/2013 | 2013 | August | late.summer.autumn | 1 | 200 | 1 | 1 | yes | yes | never | sheds.3to4 | 3.533568905 | 1 | 2 | 0.566 | 446.95 | 0 | 0 | 31 | 61.3 | yes | N |
| training | 137 | 31/07/2013 | 2013 | August | late.summer.autumn | 2 | 130 | 0 | 1 | yes | yes | allyear | sheds.3to4 | 2.202643172 | 1 | 1 | 0.454 | 501 | 0 | 0 | 29 | 55.2 | no | Y |
| training | 150 | 28/08/2013 | 2013 | September | late.summer.autumn | 1 | 300 | 0 | 1 | no | yes | never | sheds.3to4 | 4.385964912 | 1 | 2 | 0.456 | 365.92 | 0 | 0 | 35 | 28.6 | no | N |
| training | 151 | 28/08/2013 | 2013 | September | late.summer.autumn | 1 | 70 | 0 | 1 | no | yes | never | sheds.3to4 | 2.840909091 | 0 | 2 | 0.704 | 276.07 | 0 | 0 | 35 | 28.6 | no | N |
| training | 3 | 12/04/2012 | 2012 | April | spring | 0 | 150 | 0 | 1 | yes | yes | never | sheds.3to4 | 5.563282337 | 0 | 4 | 0.719 | 295.0853889 | 0 | 0 | 29 | 82.8 | no | Y |
| training | 8 | 16/04/2012 | 2012 | May | spring | 2 | 100 | 1 | 1 | no | yes | never | sheds.3to4 | 6.765899865 | 0 | 5 | 0.739 | 82 | 25 | 1 | 29 | 75.9 | no | Y |
| training | 9 | 16/04/2012 | 2012 | May | spring | 2 | 200 | 1 | 1 | no | yes | never | sheds.3to4 | 4.246284501 | 1 | 2 | 0.471 | 501 | 0 | 0 | 29 | 86.2 | yes | Y |
| training | 12 | 18/04/2012 | 2012 | May | spring | 1 | 230 | 1 | 1 | yes | yes | never | sheds.3to4 | 13.79310345 | 0 | 6 | 0.435 | 289.8793716 | 7 | 1 | 30 | 80 | yes | N |
| training | 15 | 23/04/2012 | 2012 | May | spring | 2 | 200 | 0 | 0 | yes | yes | sometimes | sheds.3to4 | 2.766251729 | 1 | 2 | 0.723 | 449.302368 | 0 | 0 | 30 | 76.7 | yes | N |
| training | 19 | 27/04/2012 | 2012 | May | spring | 3 | 220 | 0 | 1 | yes | yes | allyear | sheds.3to4 | 1.824817518 | 0 | 1 | 0.548 | 446.8118122 | 0 | 0 | 31 | 61.3 | no | Y |
| training | 26 | 08/05/2012 | 2012 | May | spring | 2 | 300 | 1 | 0 | yes | yes | never | sheds.3to4 | 20.40816327 | 0 | 15 | 0.735 | 189.1647198 | 21 | 1 | 29 | 41.4 | no | N |
| training | 29 | 10/05/2012 | 2012 | May | spring | 4 | 100 | 0 | 1 | no | yes | sometimes | sheds.3to4 | 4.347826087 | 0 | 3 | 0.69 | 146.9815484 | 0 | 0 | 32 | 46.9 | no | Y |
| training | 40 | 28/05/2012 | 2012 | June | summer | 3 | 350 | 1 | 1 | yes | yes | never | sheds.3to4 | 8.917197452 | 0 | 7 | 0.785 | 277.4600988 | 0 | 0 | 30 | 80 | yes | N |
| training | 46 | 14/06/2012 | 2012 | July | summer | 0 | 100 | 0 | 1 | yes | no | never | sheds.3to4 | 6.872852234 | 0 | 4 | 0.582 | 186.2 | 1 | 1 | 33 | 90.9 | no | N |
| training | 59 | 30/07/2012 | 2012 | August | late.summer.autumn | 1 | 50 | 0 | 1 | yes | no | never | sheds.3to4 | 10.60070671 | 0 | 6 | 0.566 | 78.78005611 | 2 | 1 | 29 | 79.3 | no | N |
| training | 62 | 03/08/2012 | 2012 | August | late.summer.autumn | 2 | 140 | 0 | 1 | yes | yes | never | sheds.3to4 | 4.398826979 | 0 | 3 | 0.682 | 243.1390851 | 0 | 0 | 32 | 84.4 | yes | N |
| training | 69 | 09/08/2012 | 2012 | August | late.summer.autumn | 5 | 450 | 1 | 0 | yes | yes | never | sheds.3to4 | 0 | 0 | 0 | 0.785 | 501 | 0 | 0 | 29 | 82.8 | no | N |
| training | 81 | 30/04/2013 | 2013 | May | spring | 0 | 25 | 1 | 1 | no | no | never | sheds.3to4 | 2.949852507 | 1 | 2 | 0.678 | 501 | 0 | 0 | 31 | 48.4 | no | N |
| training | 84 | 02/05/2013 | 2013 | May | spring | 3 | 350 | 1 | 1 | no | yes | sometimes | sheds.3to4 | 0 | 0 | 0 | 0.627 | 501 | 1 | 1 | 29 | 51.7 | yes | N |
| training | 89 | 05/05/2013 | 2013 | May | spring | 2 | 250 | 1 | 0 | yes | yes | allyear | sheds.3to4 | 0 | 0 | 0 | 0.565 | 501 | 0 | 0 | 30 | 50 | yes | N |
| training | 95 | 14/05/2013 | 2013 | May | spring | 2 | 400 | 1 | 0 | yes | no | never | sheds.3to4 | 4.021447721 | 1 | 3 | 0.746 | 383.69 | 0 | 0 | 30 | 40 | yes | N |
| training | 96 | 14/05/2013 | 2013 | May | spring | 2 | 110 | 0 | 1 | yes | yes | allyear | sheds.3to4 | 4.026845638 | 0 | 3 | 0.745 | 245.59 | 1 | 1 | 30 | 40 | no | N |
| training | 99 | 22/05/2013 | 2013 | June | summer | 1 | 135 | 1 | 1 | yes | yes | never | sheds.3to4 | 5.449591281 | 0 | 4 | 0.734 | 397.52 | 0 | 0 | 33 | 45.5 | yes | Y |
| training | 103 | 31/05/2013 | 2013 | June | summer | 1 | 100 | 0 | 1 | yes | yes | allyear | sheds.3to4 | 2.688172043 | 1 | 2 | 0.744 | 392.76 | 0 | 0 | 32 | 56.3 | no | N |
| training | 135 | 26/07/2013 | 2013 | August | late.summer.autumn | 0 | 150 | 1 | 1 | yes | yes | never | sheds.3to4 | 7.987220447 | 1 | 5 | 0.626 | 277.86 | 0 | 0 | 28 | 50 | yes | N |
| training | 142 | 14/08/2013 | 2013 | August | late.summer.autumn | 1 | 215 | 1 | 1 | yes | yes | never | sheds.3to4 | 6.465517241 | 0 | 3 | 0.464 | 388.46 | 1 | 1 | 35 | 48.6 | yes | N |
| training | 152 | 28/08/2013 | 2013 | September | late.summer.autumn | 3 | 250 | 0 | 1 | yes | yes | allyear | sheds.3to4 | 0 | 1 | 0 | 0.567 | 501 | 0 | 0 | 35 | 28.6 | yes | Y |
| training | 153 | 28/08/2013 | 2013 | September | late.summer.autumn | 2 | 120 | 1 | 1 | yes | yes | allyear | sheds.3to4 | 0 | 0 | 0 | 0.584 | 501 | 0 | 0 | 35 | 28.6 | yes | N |
| training | 10 | 17/04/2012 | 2012 | May | spring | 3 | 200 | 1 | 1 | yes | yes | allyear | sheds.5ormore | 5.839416058 | 0 | 4 | 0.685 | 384.9273549 | 26 | 1 | 29 | 89.7 | no | N |
| training | 16 | 24/04/2012 | 2012 | May | spring | 5 | 520 | 0 | 1 | yes | yes | allyear | sheds.5ormore | 6.369426752 | 1 | 5 | 0.785 | 202.3025914 | 0 | 0 | 29 | 75.9 | no | N |
| training | 20 | 30/04/2012 | 2012 | May | spring | 3 | 200 | 1 | 1 | yes | yes | never | sheds.5ormore | 6.45994832 | 0 | 5 | 0.774 | 501 | 0 | 0 | 30 | 53.3 | no | Y |
| training | 28 | 10/05/2012 | 2012 | May | spring | 3 | 195 | 0 | 1 | no | yes | never | sheds.5ormore | 7.766990291 | 1 | 4 | 0.515 | 110.374291 | 17 | 1 | 31 | 45.2 | no | N |
| training | 51 | 28/06/2012 | 2012 | July | summer | 3 | 600 | 0 | 1 | yes | yes | never | sheds.5ormore | 0 | 0 | 0 | 0.542 | 501 | 0 | 0 | 29 | 79.3 | no | N |
| training | 70 | 13/08/2012 | 2012 | August | late.summer.autumn | 2 | 150 | 1 | 1 | yes | yes | sometimes | sheds.5ormore | 19.26444834 | 0 | 11 | 0.571 | 100.6999021 | 3 | 1 | 29 | 62.1 | yes | Y |
| training | 75 | 24/08/2012 | 2012 | September | late.summer.autumn | 2 | 200 | 0 | 1 | yes | yes | never | sheds.5ormore | 1.353179973 | 0 | 1 | 0.739 | 501 | 0 | 0 | 31 | 35.5 | yes | N |
| training | 83 | 03/05/2013 | 2013 | May | spring | 3 | 260 | 1 | 1 | yes | yes | allyear | sheds.5ormore | 1.703577513 | 1 | 1 | 0.587 | 273.7 | 0 | 0 | 29 | 51.7 | yes | N |
| training | 87 | 08/05/2013 | 2013 | May | spring | 1 | 200 | 1 | 1 | yes | yes | allyear | sheds.5ormore | 2.89017341 | 0 | 2 | 0.692 | 273.42 | 0 | 0 | 29 | 69 | no | N |
| training | 88 | 08/05/2013 | 2013 | May | spring | 1 | 250 | 1 | 1 | yes | yes | allyear | sheds.5ormore | 2.012072435 | 1 | 1 | 0.497 | 306.71 | 28 | 1 | 30 | 50 | yes | Y |
| training | 90 | 09/05/2013 | 2013 | May | spring | 4 | 500 | 1 | 1 | yes | yes | allyear | sheds.5ormore | 5.008347245 | 0 | 3 | 0.599 | 407.19 | 27 | 1 | 29 | 62.1 | yes | Y |
| training | 127 | 10/07/2013 | 2013 | July | summer | 0 | 150 | 0 | 1 | no | no | never | sheds.5ormore | 6.276150628 | 0 | 3 | 0.478 | 249.39 | 0 | 0 | 30 | 43.3 | yes | Y |
| training | 129 | 11/07/2013 | 2013 | July | summer | 1 | 400 | 1 | 1 | yes | yes | allyear | sheds.5ormore | 1.455604076 | 1 | 1 | 0.687 | 220.33 | 0 | 0 | 29 | 48.3 | yes | N |
| training | 143 | 14/08/2013 | 2013 | August | late.summer.autumn | 1 | 200 | 1 | 1 | yes | yes | never | sheds.5ormore | 0 | 1 | 0 | 0.525 | 501 | 2 | 1 | 35 | 40 | yes | N |
| training | 145 | 16/08/2013 | 2013 | September | late.summer.autumn | 2 | 300 | 1 | 1 | yes | yes | never | sheds.5ormore | 9.900990099 | 0 | 6 | 0.606 | 198.31 | 6 | 1 | 35 | 60 | yes | Y |
| training | 154 | 30/08/2013 | 2013 | September | late.summer.autumn | 3 | 150 | 1 | 1 | yes | yes | never | sheds.5ormore | 1.727115717 | 0 | 1 | 0.579 | 501 | 0 | 0 | 32 | 56.3 | yes | Y |
| training | 1 | 11/04/2012 | 2012 | April | spring | 1 | 200 | 1 | 0 | no | yes | sometimes | sheds.5ormore | 10.02004008 | 1 | 5 | 0.499 | 94.118203 | 30 | 1 | 30 | 93.3 | no | N |
| training | 4 | 12/04/2012 | 2012 | May | spring | 4 | 330 | 1 | 1 | yes | yes | allyear | sheds.5ormore | 5.540166205 | 0 | 4 | 0.722 | 214.1672347 | 5 | 1 | 29 | 82.8 | no | N |
| training | 17 | 25/04/2012 | 2012 | May | spring | 3 | 110 | 1 | 1 | yes | yes | never | sheds.5ormore | 0 | 0 | 0 | 0.59 | 501 | 0 | 0 | 33 | 60.6 | yes | N |
| training | 36 | 18/05/2012 | 2012 | June | summer | 6 | 500 | 1 | 1 | yes | yes | allyear | sheds.5ormore | 1.44092219 | 0 | 1 | 0.694 | 501 | 0 | 0 | 31 | 48.4 | yes | Y |
| training | 44 | 30/05/2012 | 2012 | June | summer | 2 | 150 | 0 | 1 | no | yes | never | sheds.5ormore | 14.1025641 | 0 | 11 | 0.78 | 245.5373826 | 3 | 1 | 28 | 85.7 | yes | N |
| training | 48 | 20/06/2012 | 2012 | July | summer | 1 | 130 | 0 | 1 | yes | yes | never | sheds.5ormore | 6.896551724 | 0 | 5 | 0.725 | 496.5611011 | 0 | 0 | 30 | 90 | no | Y |
| training | 67 | 08/08/2012 | 2012 | August | late.summer.autumn | 3 | 750 | 1 | 1 | yes | yes | allyear | sheds.5ormore | 7.575757576 | 1 | 5 | 0.66 | 51.04234612 | 0 | 0 | 29 | 69 | yes | N |
| training | 82 | 01/05/2013 | 2013 | May | spring | 1 | 330 | 1 | 1 | yes | yes | allyear | sheds.5ormore | 2.663115846 | 0 | 2 | 0.751 | 463.16 | 0 | 0 | 28 | 46.4 | yes | N |
| training | 98 | 21/05/2013 | 2013 | June | summer | 2 | 350 | 1 | 1 | yes | yes | allyear | sheds.5ormore | 2.547770701 | 1 | 2 | 0.785 | 288.85 | 0 | 0 | 35 | 51.4 | yes | N |
| training | 115 | 27/06/2013 | 2013 | July | summer | 1 | 250 | 0 | 1 | yes | yes | allyear | sheds.5ormore | 3.821656051 | 0 | 3 | 0.785 | 315.68 | 10 | 1 | 29 | 20.7 | no | N |
| training | 118 | 27/06/2013 | 2013 | July | summer | 2 | 320 | 1 | 1 | yes | yes | allyear | sheds.5ormore | 6.369426752 | 0 | 5 | 0.785 | 96.35 | 18 | 1 | 29 | 20.7 | yes | N |
| training | 131 | 16/07/2013 | 2013 | July | summer | 2 | 120 | 0 | 1 | no | yes | never | sheds.5ormore | 40.74074074 | 0 | 11 | 0.27 | 218.62 | 22 | 1 | 31 | 48.4 | no | N |
| training | 133 | 18/07/2013 | 2013 | August | late.summer.autumn | 3 | 230 | 1 | 1 | yes | yes | allyear | sheds.5ormore | 10.94391245 | 0 | 8 | 0.731 | 302.9 | 4 | 1 | 29 | 51.7 | yes | N |
| training | 148 | 21/08/2013 | 2013 | September | late.summer.autumn | 2 | 400 | 1 | 1 | yes | yes | allyear | sheds.5ormore | 7.776049767 | 0 | 5 | 0.643 | 409.59 | 0 | 0 | 35 | 40 | yes | N |
| training | 27 | 09/05/2012 | 2012 | May | spring | 3 | 300 | 0 | 1 | yes | yes | never | sheds.5ormore | 16.50618982 | 0 | 12 | 0.727 | 303.5476814 | 22 | 1 | 28 | 35.7 | no | N |
| training | 144 | 15/08/2013 | 2013 | September | late.summer.autumn | 5 | 1100 | 1 | 1 | yes | yes | allyear | sheds.5ormore | 3.305785124 | 1 | 2 | 0.605 | 320.25 | 0 | 0 | 36 | 58.3 | yes | N |
| training | 155 | 02/10/2013 | 2013 | October | late.summer.autumn | 4 | 400 | 1 | 1 | yes | yes | allyear | sheds.5ormore | 8.048289738 | 1 | 4 | 0.497 | 256.54 | 0 | 0 | 34 | 79.4 | yes | N |
| training | 31 | 14/05/2012 | 2012 | May | spring | 2 | 100 | 1 | 1 | yes | yes | never | sheds.5ormore | 10.83032491 | 0 | 6 | 0.554 | 307.2036023 | 4 | 1 | 32 | 50 | no | Y |
| training | 64 | 06/08/2012 | 2012 | August | late.summer.autumn | 3 | 300 | 1 | 1 | no | yes | never | sheds.5ormore | 1.481481481 | 1 | 1 | 0.675 | 124.6596464 | 11 | 1 | 31 | 77.4 | yes | N |
| training | 76 | 17/04/2013 | 2013 | May | spring | 5 | 220 | 1 | 1 | yes | yes | allyear | sheds.5ormore | 0 | 0 | 0 | 0.632 | 501 | 1 | 1 | 29 | 31 | yes | Y |
| training | 5 | 13/04/2012 | 2012 | April | spring | 6 | 300 | 1 | 1 | yes | yes | allyear | sheds.5ormore | 0 | 0 | 0 | 0.785 | 501 | 0 | 0 | 29 | 93.1 | no | Y |
| training | 21 | 30/04/2012 | 2012 | May | spring | 4 | 420 | 0 | 1 | yes | yes | never | sheds.5ormore | 4.901960784 | 1 | 3 | 0.612 | 235.6429781 | 19 | 1 | 30 | 50 | no | N |
| training | 61 | 02/08/2012 | 2012 | August | late.summer.autumn | 3 | 300 | 1 | 1 | yes | yes | never | sheds.5ormore | 1.488095238 | 0 | 1 | 0.672 | 326.5020975 | 0 | 0 | 29 | 86.2 | yes | N |
| training | 14 | 23/04/2012 | 2012 | May | spring | 3 | 200 | 0 | 1 | yes | yes | allyear | sheds.5ormore | 9.070294785 | 1 | 4 | 0.441 | 473.2254572 | 0 | 0 | 30 | 76.7 | yes | N |
| test | 156 | NA | NA | NA | NA | 2 | 91 | NA | yes | NA | NA | NA | sheds.upto.2 | 0 | NA | 0 | 0.785 | 501 | 0 | 0 | 378 | NA | NA | NA |
| test | 157 | NA | NA | NA | NA | 4 | 125 | NA | no | NA | NA | NA | sheds.upto.2 | 0 | NA | 0 | 0.785 | 501 | 1 | 1 | 370 | NA | NA | NA |
| test | 158 | NA | NA | NA | NA | 1 | 135 | NA | yes | NA | NA | NA | sheds.3to4 | 7.643312102 | NA | 6 | 0.785 | 159 | 248 | 1 | 394 | NA | NA | NA |
| test | 159 | NA | NA | NA | NA | 1 | 171 | NA | yes | NA | NA | NA | sheds.3to4 | 1.27388535 | NA | 1 | 0.785 | 501 | 1 | 1 | 366 | NA | NA | NA |
| test | 160 | NA | NA | NA | NA | 2 | 130 | NA | yes | NA | NA | NA | sheds.3to4 | 5.095541401 | NA | 4 | 0.785 | 419 | 258 | 1 | 366 | NA | NA | NA |
| test | 161 | NA | NA | NA | NA | 2 | 240 | NA | yes | NA | NA | NA | sheds.5ormore | 0 | NA | 0 | 0.785 | 501 | 0 | 0 | 378 | NA | NA | NA |
| test | 162 | NA | NA | NA | NA | 2 | 255 | NA | no | NA | NA | NA | sheds.3to4 | 20.38216561 | NA | 16 | 0.785 | 51 | 239 | 1 | 377 | NA | NA | NA |
| test | 163 | NA | NA | NA | NA | 4 | 202 | NA | no | NA | NA | NA | sheds.3to4 | 11.46496815 | NA | 9 | 0.785 | 156 | 0 | 0 | 378 | NA | NA | NA |
| test | 164 | NA | NA | NA | NA | 4 | 218 | NA | yes | NA | NA | NA | sheds.5ormore | 11.46496815 | NA | 9 | 0.785 | 351 | 52 | 1 | 366 | NA | NA | NA |
| test | 165 | NA | NA | NA | NA | 2 | 96 | NA | yes | NA | NA | NA | sheds.upto.2 | 1.27388535 | NA | 1 | 0.785 | 187 | 1 | 1 | 366 | NA | NA | NA |
| test | 166 | NA | NA | NA | NA | 5 | 347 | NA | yes | NA | NA | NA | sheds.5ormore | 0 | NA | 0 | 0.785 | 501 | 0 | 0 | 378 | NA | NA | NA |
| test | 167 | NA | NA | NA | NA | 1 | 94 | NA | no | NA | NA | NA | sheds.upto.2 | 1.27388535 | NA | 1 | 0.785 | 193 | 1 | 1 | 387 | NA | NA | NA |
| test | 168 | NA | NA | NA | NA | 2 | 273 | NA | yes | NA | NA | NA | sheds.3to4 | 0 | NA | 0 | 0.785 | 501 | 0 | 0 | 378 | NA | NA | NA |
| test | 169 | NA | NA | NA | NA | 2 | 455 | NA | no | NA | NA | NA | sheds.3to4 | 3.821656051 | NA | 3 | 0.785 | 306 | 0 | 0 | 378 | NA | NA | NA |
| test | 170 | NA | NA | NA | NA | 1 | 82 | NA | yes | NA | NA | NA | sheds.3to4 | 3.821656051 | NA | 3 | 0.785 | 435 | 1 | 1 | 387 | NA | NA | NA |
| test | 171 | NA | NA | NA | NA | 3 | 578 | NA | no | NA | NA | NA | sheds.3to4 | 0 | NA | 0 | 0.785 | 501 | 3 | 1 | 367 | NA | NA | NA |
| test | 172 | NA | NA | NA | NA | 2 | 111 | NA | yes | NA | NA | NA | sheds.5ormore | 1.27388535 | NA | 1 | 0.785 | 256 | 0 | 0 | 378 | NA | NA | NA |
| test | 173 | NA | NA | NA | NA | 2 | 52 | NA | yes | NA | NA | NA | sheds.3to4 | 0 | NA | 0 | 0.785 | 501 | 0 | 0 | 378 | NA | NA | NA |
| test | 174 | NA | NA | NA | NA | 2 | 333 | NA | yes | NA | NA | NA | sheds.3to4 | 0 | NA | 0 | 0.785 | 501 | 0 | 0 | 378 | NA | NA | NA |
| test | 175 | NA | NA | NA | NA | 4 | 195 | NA | yes | NA | NA | NA | sheds.3to4 | 1.27388535 | NA | 1 | 0.785 | 485 | 10 | 1 | 377 | NA | NA | NA |
| test | 176 | NA | NA | NA | NA | 3 | 97 | NA | yes | NA | NA | NA | sheds.3to4 | 1.27388535 | NA | 1 | 0.785 | 442 | 0 | 0 | 378 | NA | NA | NA |
| test | 177 | NA | NA | NA | NA | 7 | 673 | NA | no | NA | NA | NA | sheds.upto.2 | 8.917197452 | NA | 7 | 0.785 | 172 | 1 | 1 | 366 | NA | NA | NA |
| test | 178 | NA | NA | NA | NA | 3 | 46 | NA | no | NA | NA | NA | sheds.upto.2 | 6.369426752 | NA | 5 | 0.785 | 54 | 6 | 1 | 396 | NA | NA | NA |
| test | 179 | NA | NA | NA | NA | 2 | 199 | NA | yes | NA | NA | NA | sheds.3to4 | 0 | NA | 0 | 0.785 | 501 | 0 | 0 | 378 | NA | NA | NA |
| test | 180 | NA | NA | NA | NA | 1 | 388 | NA | no | NA | NA | NA | sheds.3to4 | 3.821656051 | NA | 3 | 0.785 | 142 | 0 | 0 | 378 | NA | NA | NA |
| test | 181 | NA | NA | NA | NA | 0 | 181 | NA | yes | NA | NA | NA | sheds.5ormore | 1.27388535 | NA | 1 | 0.785 | 496 | 0 | 0 | 378 | NA | NA | NA |
| test | 182 | NA | NA | NA | NA | 1 | 78 | NA | yes | NA | NA | NA | sheds.upto.2 | 2.547770701 | NA | 2 | 0.785 | 222 | 29 | 1 | 365 | NA | NA | NA |
| test | 183 | NA | NA | NA | NA | 2 | 109 | NA | yes | NA | NA | NA | sheds.3to4 | 1.27388535 | NA | 1 | 0.785 | 492 | 0 | 0 | 378 | NA | NA | NA |
| test | 184 | NA | NA | NA | NA | 3 | 426 | NA | yes | NA | NA | NA | sheds.3to4 | 0 | NA | 0 | 0.785 | 501 | 0 | 0 | 378 | NA | NA | NA |
| test | 185 | NA | NA | NA | NA | 1 | 210 | NA | yes | NA | NA | NA | sheds.5ormore | 0 | NA | 0 | 0.785 | 501 | 0 | 0 | 388 | NA | NA | NA |
| test | 186 | NA | NA | NA | NA | 5 | 371 | NA | yes | NA | NA | NA | sheds.5ormore | 7.643312102 | NA | 6 | 0.785 | 177 | 42 | 1 | 388 | NA | NA | NA |
| test | 187 | NA | NA | NA | NA | 1 | 84 | NA | yes | NA | NA | NA | sheds.3to4 | 0 | NA | 0 | 0.785 | 501 | 0 | 0 | 378 | NA | NA | NA |
| test | 188 | NA | NA | NA | NA | 4 | 107 | NA | yes | NA | NA | NA | sheds.5ormore | 11.46496815 | NA | 9 | 0.785 | 288 | 30 | 1 | 393 | NA | NA | NA |
| test | 189 | NA | NA | NA | NA | 1 | 175 | NA | yes | NA | NA | NA | sheds.upto.2 | 1.27388535 | NA | 1 | 0.785 | 501 | 0 | 0 | 378 | NA | NA | NA |
| test | 190 | NA | NA | NA | NA | 2 | 68 | NA | yes | NA | NA | NA | sheds.upto.2 | 7.643312102 | NA | 6 | 0.785 | 228 | 127 | 1 | 366 | NA | NA | NA |
| test | 191 | NA | NA | NA | NA | 3 | 88 | NA | yes | NA | NA | NA | sheds.3to4 | 0 | NA | 0 | 0.785 | 501 | 2 | 1 | 378 | NA | NA | NA |
| test | 192 | NA | NA | NA | NA | 2 | 311 | NA | yes | NA | NA | NA | sheds.3to4 | 1.27388535 | NA | 1 | 0.785 | 501 | 30 | 1 | 384 | NA | NA | NA |
| test | 193 | NA | NA | NA | NA | 2 | 205 | NA | yes | NA | NA | NA | sheds.3to4 | 0 | NA | 0 | 0.785 | 501 | 0 | 0 | 378 | NA | NA | NA |
| test | 194 | NA | NA | NA | NA | 1 | 90 | NA | no | NA | NA | NA | sheds.upto.2 | 3.821656051 | NA | 3 | 0.785 | 290 | 0 | 0 | 378 | NA | NA | NA |
| test | 195 | NA | NA | NA | NA | 4 | 181 | NA | yes | NA | NA | NA | sheds.5ormore | 0 | NA | 0 | 0.785 | 501 | 0 | 0 | 378 | NA | NA | NA |
